# The largest prehistoric mound in Europe is the Bronze-Age Hill of Udine (Italy) and legend linked its origin to Attila the Hun

**DOI:** 10.1038/s41598-023-35175-8

**Published:** 2023-05-31

**Authors:** A. Fontana, G. Vinci, L. Ronchi, A. Mocchiutti, G. Muscio, P. Visentini, M. Bassetti, M. D. Novellino, F. Badino, G. Musina, S. Bonomi

**Affiliations:** 1 Department of Geosciences, University of Padova, Padua, Italy; 2 Department of History and Cultural Heritage, University of Siena, Siena, Italy; 3 Geomok Srl, Udine, Italy; 4 Museo Friulano di Storia Naturale, Comune di Udine, Udine, Italy; 5 Cora Società Archeologica Srl, Trento, Italy; 6 CNR-IGAG, Laboratory of Palinology and Paleoecology, Milan, Italy; 7 Soprintendenza Archeologia belle arti e paesaggio del Friuli Venezia Giulia, Trieste, Italy

**Keywords:** Archaeology, Geomorphology

## Abstract

Prehistoric monuments often constitute evident landmarks and sometimes, after falling into disuse, fascinated local people enough to stimulate speculations about their origin over time. According to legend, the Hill of Udine (NE Italy) was built by Attila the Hun’s soldiers, but its origin (natural or anthropogenic) has been debated until now. Our research analyzed five new 40-m long stratigraphic cores, investigating for the first time the total thickness of the hill and compared the data with the available archaeological information. Moreover, we considered other hills and mounds in northern Italy and other European regions where folklore traditions relate their origin to Attila. The geoarchaeological and ethnographic data prove that the Hill of Udine is a Bronze Age anthropogenic mound erected between 1400 and 1150 BCE and that, later, folklore has transformed the ancestral memory of its origin into legend. By measuring 30 m in height and over 400,000 m^3^ in volume, the flat-topped hill is the largest prehistoric mound in Europe. This discovery reveals unprecedented skills in earth construction and confirms significant anthropogenic modifications of the environment during Bronze Age.

## Introduction

Prehistoric monuments are archaeological structures characterized by a strong symbolic meaning or imposing dimensions and represent distinctive features of many ancient societies^1^. Their erection implied conspicuous investment of human and material resources, involving important socio-economic processes^2,3^.

Large artificial constructions are documented in the Middle East already at the Pleistocene/Holocene transition by the astonishing complex of Göbekli Tepe^4^, but they are widely attested in most prehistoric communities around the globe, as well-known examples of megaliths, pyramids and earthen mounds spread around Europe, Asia and America show [e.g.^5–7^].

**Figure 1:**
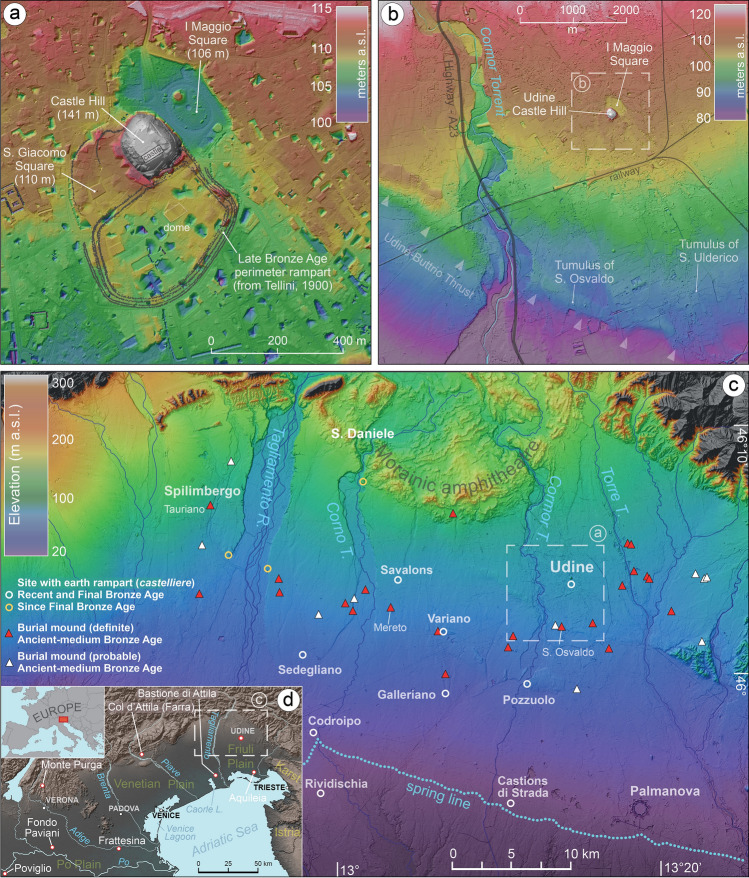
Digital Terrain Model (DTM) of the study area at local and regional scale, with indication of the sites mentioned in the text. In (**a**) and (**b**) the isolated Udine Castle Hill and the depression of I Maggio Square are evident and are not related to any surface deformation, whereas in (**b**) the arrows highlight the ridge formed by the blind tectonic thrust of Udine-Buttrio.

At global scale, human-induced modifications on the environment appear to have been widespread from around 1000 BCE^8^ and prehistoric monuments, whereas sometimes strongly degraded through millennia, are often a tangible product of these anthropogenic activities. Often, these artificial landforms represent among the rare remnants of complex cultural past landscapes largely disappeared and, generally, are highly visible landmarks over time. Sometimes, the popular interpretation of their origin fed the production of legends, folktales and toponyms, which transformed the memory of the past^9,10^. One of these places is in Udine, in the alluvial plain of NE Italy, where the city center is dominated by a 30-m high isolated hill, the Colle del Castello (Castle Hill), that, according to the popular legend, was built by Attila the Hun’s soldiers. The Huns used their helmets as buckets for the erection of a mound for allowing their leader to enjoy the spectacle of the Roman city of Aquileia in flames after the siege of 452 AD (Fig. 1). The odd location of this isolated relief has attracted the interest of the geologists since the first modern scholars but, to date, the origin of the hill remains unclear and its anthropogenic and natural components have not been yet quantified^11–13^. With an interdisciplinary approach, which integrates the geoarchaeological, archaeological and ethnographic evidence, we analyzed the characteristics of this hill by framing this research with known mounds at regional and European scale.

In Bronze Age Europe, monumental stone and earthwork structures are among the most impressive constructions^14^ and, as for alluvial environments, a remarkable example of earthworks is represented by the Terramare settlements of the Po Plain, in Northern Italy, dating back to Middle and Recent Bronze Age (MBA and RBA, ca. 1650–1150 BCE; Supplementary Table S1). These settlements were encircled by ditches and embankments, functioning both as defensive and water-management structures^15,16^. The Terramare settlement system lasted until the last part of the RBA (around 1200 BC), when it collapsed as a result of a major crisis caused by a range of ecological, economic and social factors, affecting most part of Northern Italy^17^, which was coeval with the collapse of many societies in the Eastern Mediterranean^18,19^.

Another area of Northern Italy rich in prehistoric earthworks is the Friuli Plain, where individual burial mounds (tumuli) built of gravel and earth spread from the Early Bronze Age^20^. From the MBA, similar earthwork techniques were widely adopted by communities to protect their settlements, locally known as castellieri, with imposing defensive ramparts^21^. Most of these fortified sites lasted through the Final Bronze Age (FBA) and into the Iron Age, thus surviving the collapse of the Terramare^22^. Their surface area ranges between 1.5 and 6 ha, but they are by far surpassed by the settlement of Udine, that covered an area of over 20 ha [Fig. 1a;^23^].

Since the sixteenth century CE, the origin of the hill of Udine has been debated among local scholars. In describing the hill, most of them also noticed that it is flanked by a large depression to its north-eastern side, nowadays corresponding to the I Maggio Square, which was partly occupied by a shallow lake until the nineteenth century (Fig. 1a). In 1900, Achille Tellini was the first to address the question from a modern geological perspective and suggested that a significant part of the hill was artificial, built with the sediment quarried from this basin. The creation of a large artificial pond in the center of villages was a widespread practice in the gravelly part of the Friulian Plain until the end of nineteenth century^24^. These structures were used for watering animals and people, because the groundwater table is largely unreachable as lying several tens of meters below the surface. In the twentieth century, the idea that a natural relief formed the core of the hill became dominant and was generally related to the likely presence of mid-Pleistocene morainic deposits or older fluvial conglomerates^25^. Moreover, in recent decades, the hypothesis that the hill was formed as a result of tectonic processes become widely accepted, on the grounds of comparisons with some tectonic isolated reliefs located south of Udine [Fig. 1b and c;^13,26^]. Thus, the idea that the inner sector of the hill was largely natural was held to be one of the main reasons for the origin of the pre-Roman fortified settlement of Udine^27,28^.

## Results

### Stratigraphic and archaeological data

The first written version of the legend of the construction of the Udine Castle Hill by Attila the Hun’s soldiers back to the twelfth century, when it was recorded by the chronicler Godfrey of Viterbo and Otto, bishop of Freising^11^. Interestingly, both these sources cast doubt on Attila the Hun story, presenting the alternative hypothesis that, instead, the hill was constructed by Julius Cesar. Up to modern times, several historians and chroniclers proposed their own explanation about the formation of the hill, sustaining its natural, fully-artificial or mixed origin, without providing any field data (see Supplementary information).

**Figure 2:**
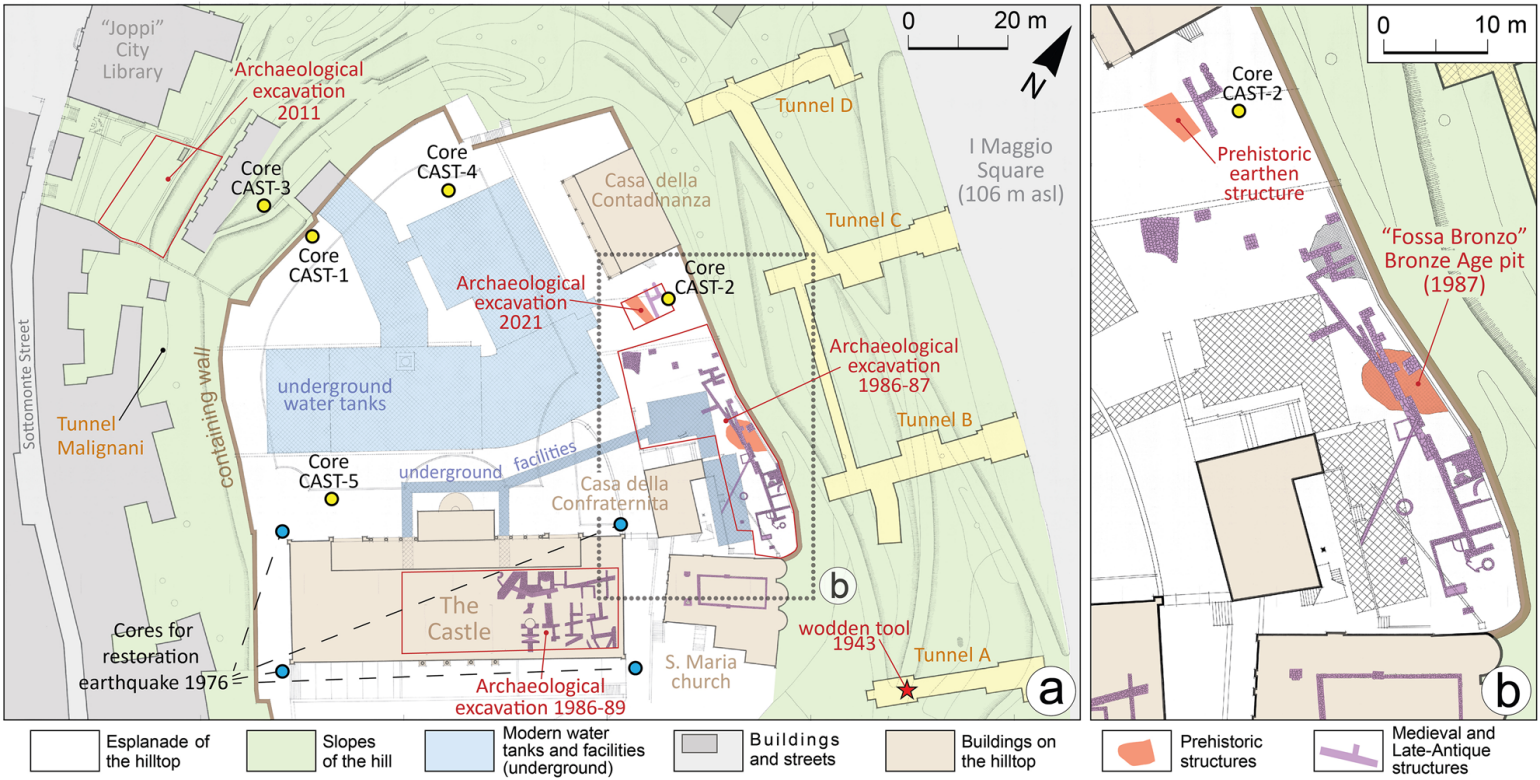
Detailed map of the hill of Udine with indications of the investigations and archaeological findings. Map of archaeological structures according to Buora^28^. The new stratigraphic cores drilled from the hilltop are indicated in yellow, while the blue dots are the geotechnical cores carried out in 1976 to restore the castle palace after the severe earthquakes that occurred that year. The red star indicates the point of the tunnel A, where a wooden tool had been found in 1943, and we radiocarbon dated it. The map was drawn with software Adobe Illustrator (www.adobe.com).

**Figure 3:**
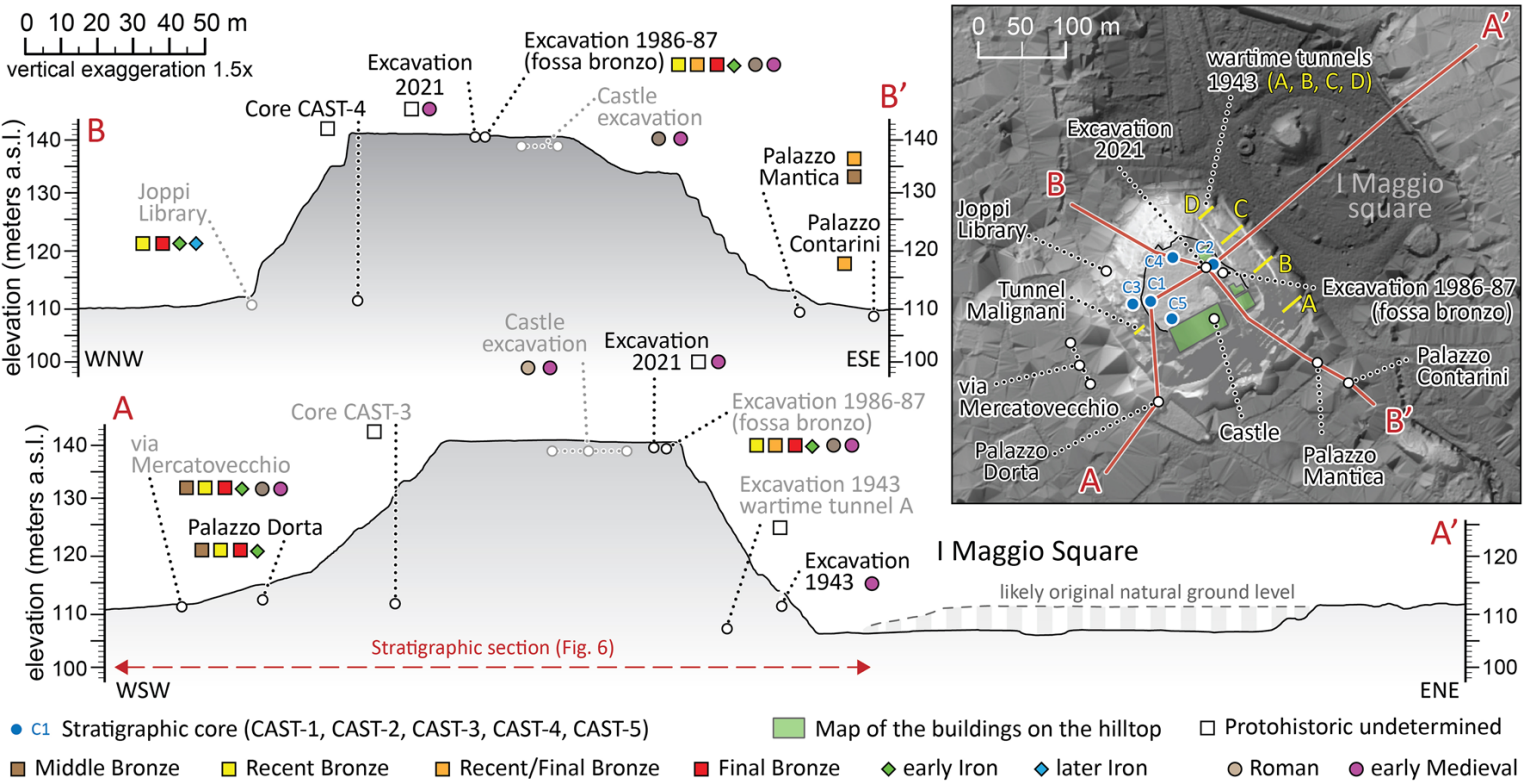
Topographic profiles of the Hill of Udine obtained by LiDAR data with indications of the stratigraphic cores and archaeological excavations. Profile A-A’ clearly shows the depression now occupied by I Maggio Square, originally occupied by a small lake, drained in the nineteenth Century.

**Figure 4:**
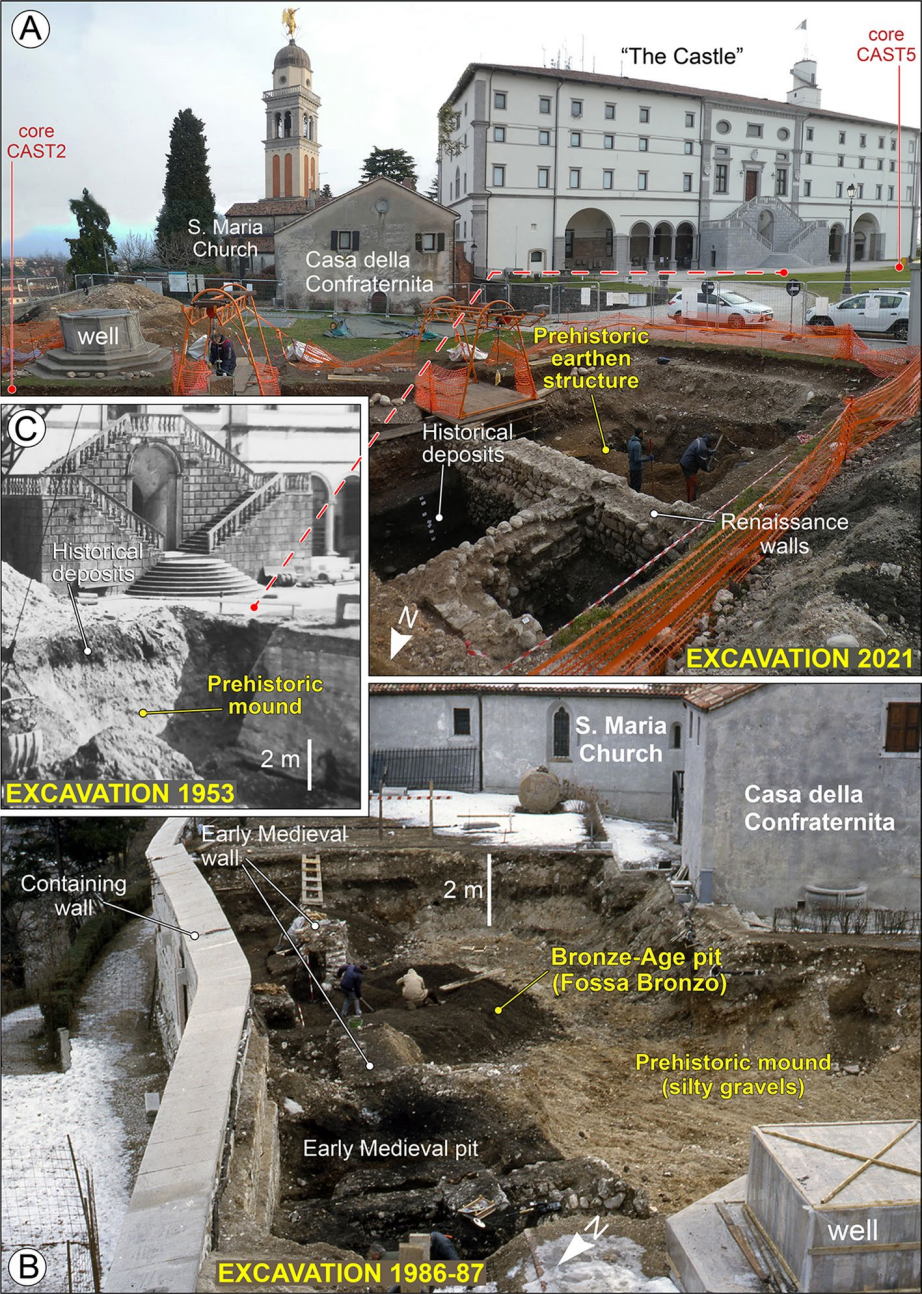
Selection of pictures of the archaeological and stratigraphic excavations carried out on the hill of Udine. (**a**) Investigations carried out between October and December 2021 identified the sub-superficial occurrence of a prehistoric structure made of alternations of gravelly and clayey lenses (picture by A. Fontana). This area is almost in continuity with the zone depicted in image (**b**) (Archives of Civici Musei of Udine), where excavation of 1987 all around the building named "Casa della Contadinanza" and near the church of S. Maria found a large pit, named “Fossa Bronzo” (Bronze-Age pit) in Buora^28^. This structure had an areal extent of about 40 m^2^ and is located at the edge of the hilltop, suggesting that it was near the slope of the mound even in ancient time. The pit was filled with an organic-rich fine matrix and thousands of potsherds. A selection of the most diagnostic fragments of pottery for chrono-typology is reported in Fig. 5 and in Supplementary Fig. S7. The fragments cover a time span between 1400 and 600 BCE, but with the majority of fragments dating to the RBA and FBA (i.e. 1350–950 BCE). (**c**) Picture taken in 1953, during the work for excavating the water tanks that now occupy most part of the esplanade of the hilltop, up to a depth of about 6 m (see Supplementary Fig. S2) (Archives of Civici Musei of Udine). The site is close to the monumental stairs of the northern entrance of the building called “the Castle”, and it has been documented that alternations of gravels and clays are present below 1.5–2 m of historical deposits rich in the organic component. A similar situation was found in core CAST-5, which is close to this site (see Fig. 4a).

Today the top of the hill features an esplanade covering about 12,000 m^2^ (Figs. 2, 3) and its immediate subsoil is occupied by several water tanks, up to 6 m deep (Fig. 2). These facilities were originally built in the first half of the twentieth century and caused the exposure of large sections that were described by Feruglio^12,25^ as documenting artificial landfills of unknown age. Despite widespread groundworks, remains of ancient structures were extensively documented in 1986–87 in the eastern sector of the hilltop, where a large excavation investigated over 500 m^2^ (Fig. 4). A number of Medieval walls and tombs and some foundation walls of Roman buildings were found below 1–2 m of Modern Age fill. Similar archaeological features were also found in the basement of the palace known as “the Castle” between 1986 and 1989^28^.

**Figure 5:**
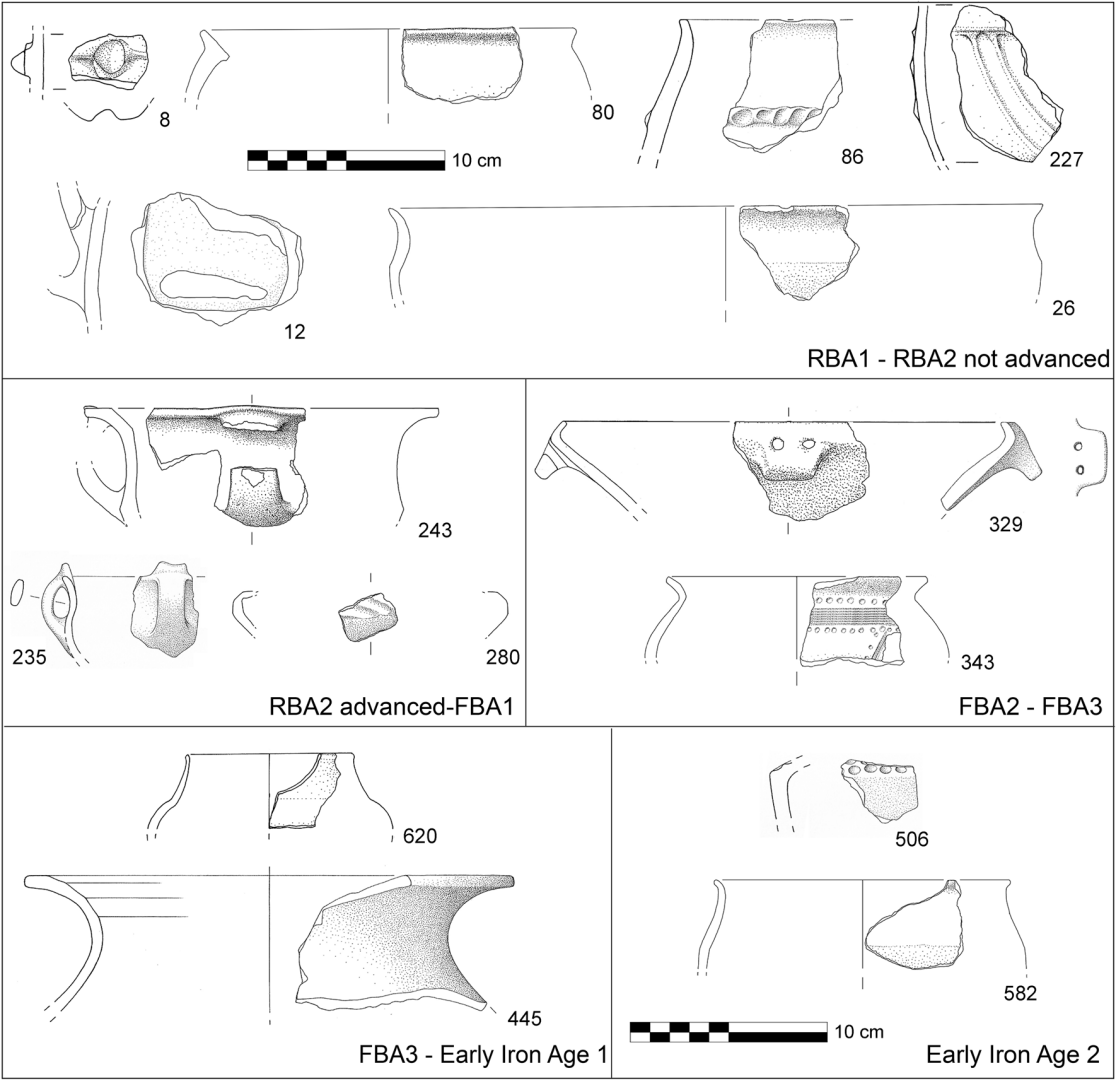
Selection of potsherds found in the pit “fossa bronzo”, investigated in 1987 during the archaeological excavation carried out along the eastern margin of the hilltop of Udine Castle Hill. The different typo-chronological phases are separated according to G. Tasca^29^ and the numbers refer to the catalog of fragments published by him^29^.

**Figure 6:**
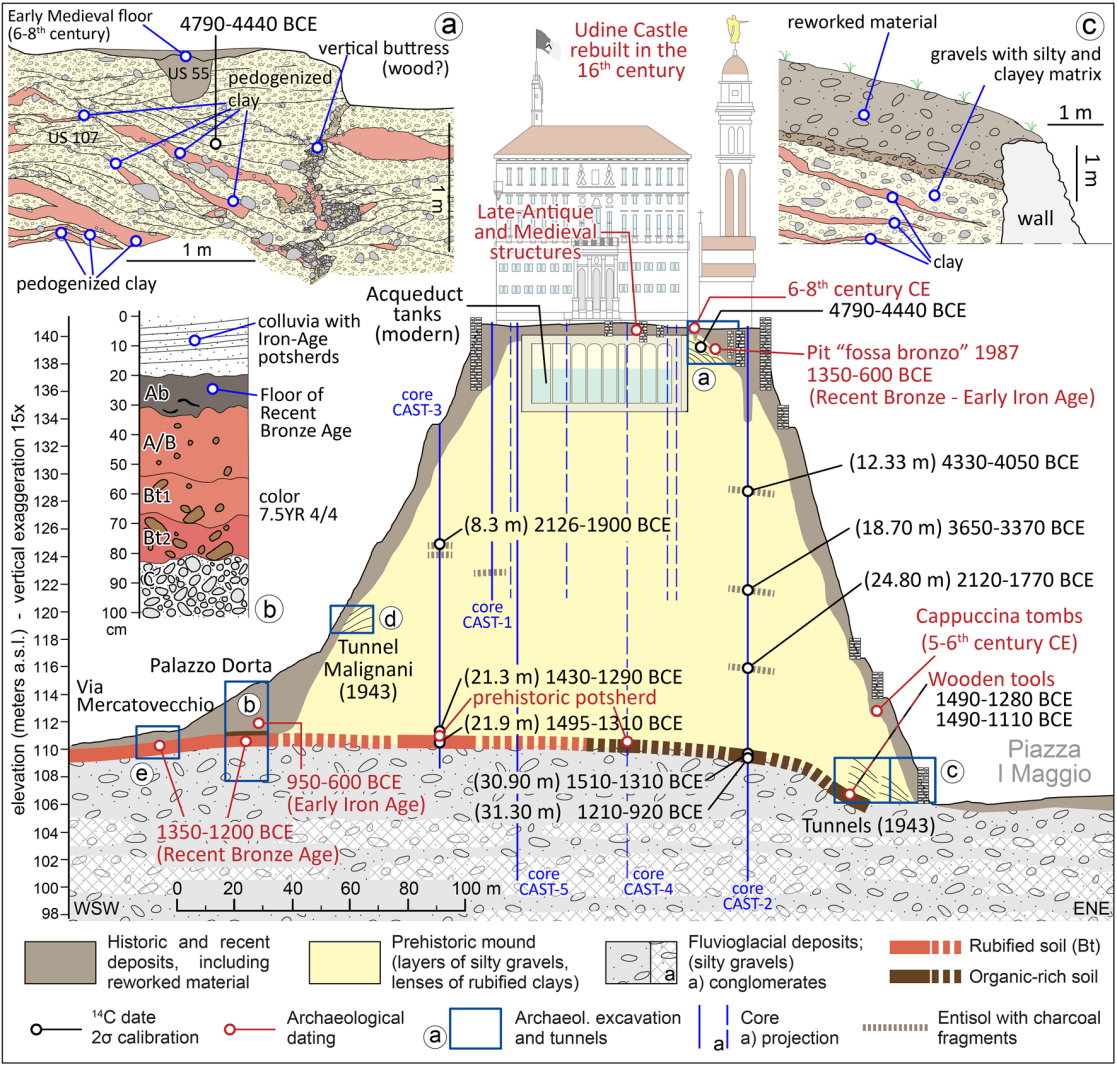
Stratigraphic section of the Udine mound with indications of cores, tunnels and archaeological excavations. The trace of the section is shown in Fig. 3 as A–A’. (**a**) 2021 archaeological excavation (test trench of 12 × 8 m). The excavation reached a depth of 3 m and enlightened floors, walls and two tombs dating between the fifth and eighth centuries CE, found just below the present ground surface; the section in the upper box shows the traces of a vertical buttress closely paralleled by the so-called “gabions”, forming the wooden framework of those earth embankments. (**b**) 2020 archaeological excavation under Palazzo Dorta. The investigation uncovered the floor of a hut of RBA dating to about 1300–1200 BCE, under 4 m of later deposits starting with a gravelly colluvium containing Iron Age potsherds and likely originating from the hill^30^. The RBA deposit overlays the original natural topography; (**c**) 1943 air-raid shelter tunnels, according to data in Someda De Marco, where a wooden tool was found^27^ and radiocarbon dated to RBA; the section in the upper box shows the stratigraphic sequence characterized by inclined successions of gravels and clayey lenses; (**d**) tunnel “Malignani” dug in 1943 into the western flank of the hill, starting at about 10.3 m above the ground level of the neighboring city center. It was dug 8.5 m horizontally into the hill and, in this case too, the stratigraphic sequence was characterized by inclined successions of gravels and clayey lenses^27^; a detailed section is not available (cf.^27^), see the text for details; (**e**) Archaeological excavation in Via Mercatovecchio; the detailed section is not displayed in this figure, see the text for details^23,30^.

A significant find was a pit of ca. 40 m^2^, named “fossa bronzo”, containing thousands of potsherds of different cultural phases, which have been recently studied in detail (Figs. 2, 3, 4 and 5). The pit can be interpreted as an erosive gully filled with an organic-rich silty matrix and potsherds. The oldest finds date to the transition between MBA to RBA^29^. Anyhow, according to the typo-chronology assemblages, the findings span from Bronze Age to Early Iron Age (ca. 1350–600 BCE), with most fragments dating to RBA and FBA [Fig. 5 and Supplementary material, Fig. S5;^29^]. In 2021 a trial trench of 12 × 8 m was opened near the 1987 excavation, up to a depth of 3 m (Fig. 4, Supplementary Fig. S3). Floors, walls and two tombs dating between the fifth and eighth centuries CE were found just below the present ground surface. As visible in Fig. 4a and Supplementary Fig. S3, these features partly reworked and lay on a sequence of silty gravels, gravels and reddish pedogenized clays of 5–15 cm in thickness. Moreover, traces of a vertical buttress were documented, most likely originally made of wood and now decomposed (Fig. 6a and Supplementary Fig. S3b). The reddish fine sediments are comparable to the neo-forming clays accumulating in the Bt horizons of the surrounding alluvial plain^13^.

Core CAST-2 was drilled at the eastern boundary of the 2021 excavation (see Supplementary Data S1 for details). Below about 6 m of reworked historical debris, the core detected repeated alternations of clays and silty gravels, comparable to those documented in the 2021 excavation. Fragments of millimetric charcoals, found in rare slightly organic horizons along the sequence of alternations, have been radiocarbon dated (Fig. 6, Table 1). The obtained ages range between 4330 and 1770 years BCE (calibrated to 2σ; Table 1$$)$$, but they are in reverse chronological order. The alternation of lenses stops at a depth of 30.8 m (109.9 m asl), where an organic-rich soil was found and two fragments of charcoal collected at depths of 30.90 and 31.30 m have been radiocarbon dated to 1510–1310 BCE and 1201–920 BCE, respectively. Below the buried soil, the sediments consist of silty gravels and weakly cemented conglomerates which match with the natural fluvioglacial sediments forming the subsoil of the city center^13^. Paleobotanical investigations carried out on pollen grains extracted between 30.90 and 31.90 m of depth in core CAST-1 documented a fairly stable vegetation, with the dominance of hazel and alder, with secondary percentage of deciduous oak (Supplementary Fig. S7). On the western side, cores CAST-1 and CAST-3 reached 20 and 25 m in depth, respectively (Supplementary Figs. S1, S6) and both cores encountered a sequence that is comparable with the lithological alternation described in CAST-2 up to 30.8 m. In CAST-3 the base of this unit was found at 21.3 m-depth It sealed slightly organic silty clays with wood remains dated to 1427–1292 BCE and a pottery fragment at 21.87 m-depth, detected just above a large wood fragment dated to 1495–1310 BCE. Also in CAST-4, in the northern sector, at 31.51 m a centimetric potsherd has been found within the soil buried by alternations of silty gravels and pedogenized clays. These sediments are also documented in CAST–4, between the depths of 3.5–31.1 m and in CAST-5, near the castle palace, between 1.5 and 30 m.

**Table 1:** Radiocarbon dates available for the mound of Udine.

n	Sample Name	Code (Lab)	Material	Description	Age ^14^C (years BP)	Calibrated age 1σ (years BCE)	Calibrated age 2σ (years BCE)
1	UD_CAS2022_US107 (excavation 2021–22)	ETH-122509 (ETH Zurich)	charcoal	Excavation 2021, charcoal in gravelly lens forming the prehistoric earthen structure	5754 ± 72	4700–4500	4790–4440
2	UD CAST2-1233 (Core CAST2)	LTL21182 (CEDAD Lecce)	charcoal	Charcoal in weak organic deposit (mound sequence) at 12.33 m of depth	5364 ± 45	4330–4060	4330–4050
3	UD CAST2-1870 (Core CAST-2)	LTL21183 (CEDAD Lecce)	charcoal	Charcoal in weak organic deposit (mound sequence) at 18.70 m of depth	4771 ± 45	3640–3520	3640–3370
4	UD CAST2-2480 (Core CAST-2)	LTL21184 (CEDAD Lecce)	charcoal	Charcoal in weak organic deposit (mound sequence) at 24.80 m of depth	3580 ± 45	2020–1880	2120–1770
5	UD CAST2-3090 (Core CAST-2)	LTL21185 (CEDAD Lecce)	charcoal	Charcoal in the soil buried at the base of the mound (30.90 m of depth)	3170 ± 40	1500–1410	1510–1310
6	UD-CAST2-3130R (Core CAST-2)	LTL21081 (CEDAD Lecce)	charcoal	Charcoal in the soil buried at the base of the mound (31.30 m of depth)	2871 ± 45	1120–930	1210–920
7	CAST3-2130 (Core CAST-3)	ETH-122881 (ETH Zurich)	wood	Large wood piece found in core CAST3 at 21.30 m of depth, at the base of the landfill forming the mound and at the top of the pre-existing soil containing pottery fragments	3100 ± 23	1420–1310	1430–1290
8	CAST3-2190 (Core CAST-3)	ETH-128856 (ETH Zurich)	wood	Large wood piece found in core CAST3 at 21.90 m of depth, inside the soil buried by the mound and containing pottery fragments	3138 ± 23	1440–1310	1495–1310
9	UdCa130 (wartime Tunnel A)	DSH10625_WO (CIRCE Caserta)	wood	Archaeological wooden artefact within the buried soil at the base of the mound; found in 1943 during excavation of the wartime tunnel A, 20 m from the entrance	3117 ± 34	4700–4500	1490–1280
10	UdCa131 (wartime Tunnels, unspecified)	DSH10820_WO (CIRCE Caserta)	wood	Archaeological wooden artefact found in 1943 during excavation of the wartime tunnels, but location is unspecified	3052 ± 68	1410–1220	1490–1110

Some more data are reported respect the information included in this Table along the main text. The analyzed samples have been selected from the material collected in 2021 and 2022 from stratigraphic cores and archaeological excavations. Only samples UdCa130 and UdCa131 are different, as they are wooden archaeological tools found in 1943 during the excavation of the wartime tunnels (Supplementary Figs. S1 and S3), dug at the base of the Udine Castle Hill on its eastern side^27^. Sample UdCa130 was found in tunnel A and sample UdCal131 in another unspecified tunnel and they are preserved in the Musei Civici.

During the Second World War, in 1943, four anti-aerial tunnels were dug in the eastern flank of the hill of Udine (Figs. 2, 3). The work started on the east side at the ground level of I Maggio Square and the tunnels were dug horizontally up to 30 m into the hill. Their excavation documented the clear eastern dip of large stratigraphic units, that consisted of alternations of gravelly and clayey layers^28^. No conglomerate or bedrock lithology was encountered but a wooden artifact was found in tunnel A at 25 m from the entrance, included in an organic-rich clayey matrix; we radiocarbon dated it to 1492–1283 BCE, comparable to the age of 1454–1111 BCE obtained from another wooden remain found in another tunnel (Fig. 6). As depicted in Fig. 6c, the sequence is very similar to the anthropogenic landfill documented at the top of the hill by the 2021 excavation. The work in 1943 stopped into few weeks, when the buildings on the eastern hilltop started to suffer significant damage due to the subsidence induced by the tunneling. This strongly suggests there are no lithified units within the hill. In 1943 another bunker (Tunnel Malignani in Figs. 2, 3) was dug in the western flank of the hill, starting at about 10.3 m above the ground level of the neighboring city center (section d in Fig. 6). It was dug 8.5 m horizontally into the hill and, also in this case, the stratigraphic sequence was characterized by inclined successions of gravels and clayey lenses^27^.

At the south-western foot of the hill, near Palazzo Dorta, an archaeological excavation in 2020 uncovered the ground floor of a hut in use up to 1300–1200 BCE^30^, which overlays the original natural topography (Fig. 6b and Supplementary Fig. S4b). Thus, the pre-existing surface lies at 110 m asl and continues almost horizontally westward. It was exposed until the second part of the RBA, but it is now covered by 4 m of later deposits that are marked at the base by a gravelly colluvium which included Iron Age potsherds and likely originated from the hill^30^. The natural soil is characterized by well-developed Bt horizons, evolved in drained conditions over the fluvioglacial deposits of the Last Glacial Maximum (LGM), which characterizes vast sectors of the plain [Fig. 5B and Supplementary Fig. S4;^13^].

Conglomeratic layers have been widely documented at Udine including in I Maggio Square below 2–6 m depth^13^ and constitute impermeable layers that allow the occurrence of superficial perched water tables, fed by the local rainfall. This stratigraphic setting is likely responsible for the stagnation of water in I Maggio Square and the historical presence of a lacustrine environment (Fig. 1a).

## Discussion

### Characteristics and chronology of the mound

The available data demonstrate that the Udine Castle Hill is comprised of loose sediments, consisting of repeated alternations of silty gravels and pedogenized reddish clays for almost its entire volume. These sediments are comparable to those forming the natural deposits and soils existing in the surrounding plain, but their layering within the hill does not correspond to a natural pattern. In particular, throughout the cores and excavations, the reddish clays have been often found out of any pedogenetic sequence. Moreover, the radiocarbon samples from the stratigraphic sequence forming the hill turned out to be chronologically reversed, suggesting that the sediments had been reworked from older deposits. These data demonstrate the artificial origin of the hill, created through the accumulation of sediments that had been likely collected in the surrounding area.

The anthropogenic mound is almost 30 m high and its construction buried the previous topography, located at an elevation between 110–111 m asl. The sealed surface is in continuity with the natural soil on which most of the city is built and corresponds to the top of the LGM fluvioglacial sediments, prone to pedogenesis since about 18,000 BCE. In the soil sealed by the mound, ancient human activity is clearly documented by prehistoric artefacts found in CAST-3, CAST-4 and in wartime tunnel A, and by the disturbance on the original forest cover, as inferred through paleobotanic analyses. The radiocarbon dates available for the buried soil testify that the original surface was exposed until the end of the MBA and it is likely that earlier significant archaeological structures existed somewhere at the base of the mound. In fact, inside the area of castelliere the first significant archaeological documentation dates back to MBA^23,30–32^.

The finding of prehistoric structures in the 1987 and 2021 excavations on the hilltop, as well as the lack of evidence for brick structures during the construction of the modern water tanks, indicates that the mound had already achieved a shape somewhat similar to its current form in prehistoric times. In particular, the eastern portion already had its present morphology around 1350–1300 BCE. All the radiocarbon dates available for assessing the age terminus post-quem for the beginning of the mound erection are almost coincident, showing a median result slightly after 1400 BCE (Table 1). The only exception is represented by sample 31.30 m in core CAST-2, that is 1201–920 BCE. Its reliability is weakened by the occurrence of an age of 1510–1310 from a charcoal sampled 40 cm above.

The technique of constructing earthworks by alternating gravelly layers and clay lenses strongly recalls that adopted during the Bronze Age in the alluvial plains of northern Italy to build both the tumuli and the ramparts of terramare and castellieri settlements^15,20,33^. This building method was clearly described in Udine in the perimeter rampart excavated in Palazzo Mantica (Fig. 2 and Supplementary Fig. S8), dating to the RBA^22^.

In contrast to the tells existing in large number in Asia and Middle East, but also documented in the Danubian plains [e.g.^34–36^], the Udine Castle Hill was not formed by sedimentary units consisting of overlapping anthropogenic layers deposited through time. On the contrary, according to the available chronostratigraphic data, the Udine Castle Hill was constructed slightly after 1400 BCE and before 1300 BCE. This probably corresponds to the significant expansion of the castelliere of Udine, also signaled by the reinforcement of the perimeter rampart documented at Palazzo Mantica^31,32^.

Roughly at the same time (i.e. RBA1-RBA2 not advanced), numerous settlements in the alluvial plain of northern Italy flourished or were founded^15,21,37^. This phase is clearly documented also at several sites located in the distal plain and in the coastal sector of NE Italy; however, these were definitely abandoned before FBA, while those in the inner plain, including Udine, survived this crisis [(Fig. 1C;^16,17,21,22,37^]. In Udine the continuity of the settlement during FBA and Iron Age is documented at several locations in the city center^23,30^, as well as on the top of the mound, as testified by the findings in the pit “fossa bronzo”^31^.

**Figure 7:**
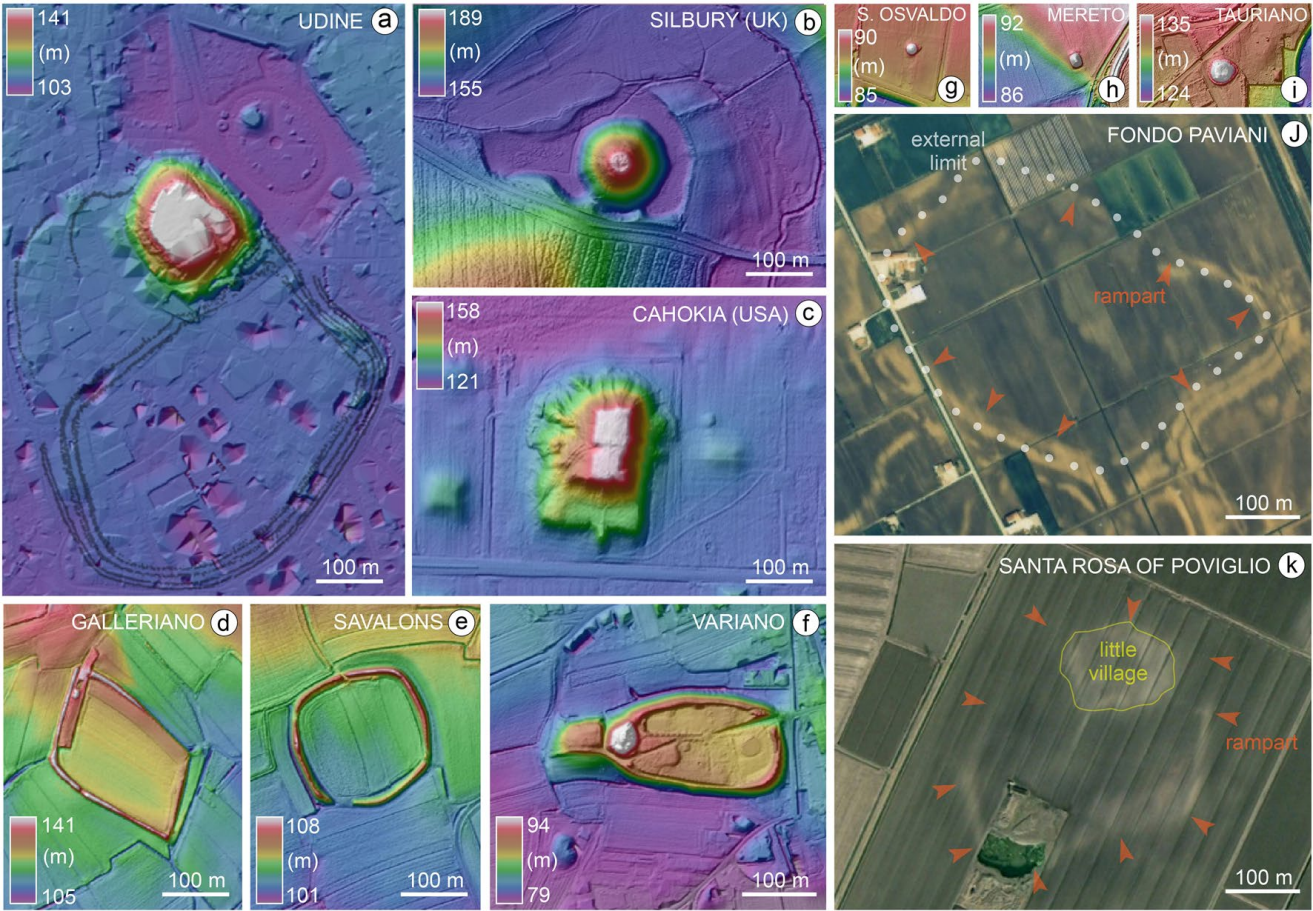
DTMs and aerial images of some of the most representative largest earth structures in the alluvial plains of northern Italy and comparison with Silbury Hill (UK) and Cahokia Monks Mound (Illinois, USA). For location of the Italian sites see Fig. 1. Figure (**j**) modified from^41^; figure (**k**) is elaborated from a satellite image of 2003 from Google Earth Maxtar Technologies 2023. The figures were produced elaborating the DEMs and images with software QGIS (https://www.qgis.org) and Adobe Illustrator (www.adobe.com).

Although later additions could have been made, shaping its current form, our data support the hypothesis that the Udine Castle Hill already existed at the beginning of RBA and had a flat-topped surface. Neither its shape or dimensions are compatible with the Bronze Age tumuli documented in the Friuli Plain (Fig. 7) and, although buildings or other structures could have been built on its large top, there is currently no evidence for any features. In fact, the original surface has been almost entirely eroded by the later occupation of the area.

At the same time, the occurrence of any natural hilly landform (e.g. hard rock formation) in the core of the mound can also be rejected, thus excluding the hypothesis of its neotectonic origin. Thus, the Castel Hill is completely artificial and was built as a monumental construction, displaying the power of the community with its size and visibility. In spite of the rather flat landscape, the pre-existing topography below the eastern sector of the mound seems to dip about 3 m towards I Maggio Square, as documented by the stratigraphy of wartime tunnels (Fig. 7c). This is likely compatible with the fluvial incision eroded by the local drainage in the post-LGM. Thus, a shallow natural depression was already present in the area, allowing the emergence of the local perched water table at the surface. This is an extraordinary situation in the gravelly sector of the Friulian Plain and may justify the interest of ancient communities for this site since remote periods. This setting is further confirmed by paleobotanic and paleopedologic data, supporting that some humid environment was previously present in the area of I Maggio Square whereas, west of it, soils were well drained.

Considering the pre-existing topography of the area, today the volume of the entire mound is about 600,000–650,000 m^3^, while the volume of the basin corresponding to I Maggio Square is assessed between 400,000 and 450,000 m^3^ (see Supplementary information and Fig. S9). The mound had a sub-circular plan since its earliest phase and the occurrence of a rather flat top already in that first period is suggested by the presence of a structure on the hilltop with Bronze and Iron Age findings. This hypothesis is further supported by the occurrence of historical deposits just in the very shallow subsoil and only along the rim of the hill. The prehistoric mound may have had a maximum volume approaching 550,000 m^3^, but, even considering a cautious assessment of its dimensions and limiting the hilltop radius at 35 m, the minimum volume ranges between 400,000 and 450,000 m^3^. The fairly good correspondence between the original volume of the mound and the sediments potentially extracted from I Maggio Square support the idea that both features are anthropogenic landforms of later Bronze Age, with a connected origin, as postulated by the first modern scholars^11,25^. Moreover, it is likely that the two actions were part of a combined project, aiming at creating a water reservoir for the settlement and using the extracted material to build a monument. It is worth noting that the excavation of the basin likely occurred during the RBA, when the site of Udine underwent a significant expansion^23^. Moreover, it matches with the onset of a severe drought occurred in northern Italy, as testified in the area of the Terramare at two main excavated settlements of Santa Rosa di Poviglio and Fondo Paviani^16,37^.

Even considering the minimum volume estimate, the size of the mound is without a parallel in any other structure of the period in northern Italy (Fig. 7) and must have been the most prominent landmark in the late prehistoric landscape of the Friuli Plain, as also confirmed by results of the gis-based visibility analysis (see Supplementary information and Fig. S10).

### Archaeology and folklore

In many folktales, both in Italy and other regions of Europe, Attila the Hun is identified as “the scourge of God” and his name has become a topos for describing the destruction and, in a wider sense, the collapse of late antique society^38^. His name has often been associated with the creation of peculiar features, both natural or anthropogenic, which mark the landscape. In Udine the king of the Huns has been used to explain the formation of the huge mound. It is likely that the legend evolved within the local community, through the reworking of the ancestral memory that the hill had a distant artificial origin.

Another mound named after Attila the Hun was located at the boundary of Caorle Lagoon, near Lugugnana (Fig. 1d, Supplementary Fig. S12). It was 5-m high, about 20 m in diameter and was levelled after the First World War. Despite its unknown age and function, people used to call it the “Bastione di Attila”, explaining its origin as part of an overnight camp of the Huns during their raid. A “Colle d’Attila” is located also at the end of the Piave River valley, near Farra di Soligo, where two isolated hillocks rise above the surrounding plain (Fig. 1d and Supplementary Fig. S11). Their position does not fit the geological context and their origin has been queried [e.g.^39^] but, according to popular tradition, they are the tumuli built by the Huns for their king.

The practice of assigning the origin of ancient mounds to the Huns is also found in the plains of Poland and Lithuania, where local people used to call Bronze and Iron Age tumuli Hunnenbette, literally “Hun beds”, considering them to date to that period^40^. South of Reims (France), near Châlons, where Attila fought with the Romans, the feature described by the local tradition as Attila’s Camp is actually a large Roman fort, already built in the first century CE^38^. In this light, at European scale, the Huns’ invasion was so traumatic to become a topos for explaining the occurrence of older archaeological artefacts whose memory had been lost.

### Relevant comparisons and social implications

Our multidisciplinary approach allowed the investigation of sedimentary bodies and ancient surfaces that are largely inaccessible with conventional archaeological excavation. In particular, as already shown in studies of the origins of other prehistoric mounds and urban contexts^42–44^, the detailed study of stratigraphic cores was key to distinguishing natural and anthropogenic sediments. Our data show that the size and shape of the Udine Castle Hill are unparalleled in later Bronze Age communities of Europe. This opens new perspectives on the study of labor organization and social complexity of these prehistoric communities. Beyond northern Italy, fortifications constructed with wooden gabions filled with rubble are attested also in central-eastern Europe at several settlements of the Lausitz culture^45^. It may be that this technique developed as a result of the unprecedented growth of network of trade and interconnections among the communities in the second part of the Bronze Age at European scale^42^.

Our data suggest that the prehistoric mound of Udine achieved its final shape during RBA and, according to the recent literature, in that phase the settlement area of the castelliere enlarged up to reaching over 20 ha^23^. This extent is much larger than the terramara of Santa Rosa di Poviglio, that had a maximum area of 7 ha, but is a reference for the RBA because of the detailed available data about its stratigraphy and plan map^15,16^. Differently, Udine is rather comparable to the largest RBA Terramare settlements of Northern Italy, as Case Cocconi, Case del Lago and Fondo Paviani^15,37^, whereas, is slightly larger than Frattesina, that was not a terramara, flourished in the FBA and reached 15 ha^47^ [Figs. 1d and 7j;^37,41,47^]. The reorganization of settlements that occurred at the end of the RBA in Friuli points to the resilience of this area to the general collapse of the Eastern Mediterranean societies in the twelfth century BCE^46^.

The mound of Udine is far larger and higher than the burial mounds and kurgans attested elsewhere in continental Europe from the Neolithic to the late Iron Age, such as in Brittany, Poland, Ukraine and Hungary, where the largest reach 12–19 m in height^48^. Otherwise, its dimensions are similar to the Neolithic mound of Silbury Hill in Wiltshire (England), built around 2400*–*2300 BCE, which has an overall volume of nearly 350,000 m^3^. As at Silbury, at Udine too the quarrying left an enormous depression in the landscape [Fig. 7b;^49^]. This comparison makes Udine Castle Hill the largest prehistoric mound of Europe.

Considering its flat-topped surface, the hill of Udine is a unicum that has no clear analogues in Europe, but it displays a convergence with the mounds conceived to host ceremonial and political structures on their top, best illustrated by the earthen platforms erected by the Mississippian culture of the SW United States between the tenth and sixteenth century CE. In particular, a relevant comparison can be established with the imposing quadrangular Monks Mound at Cahokia, in Illinois, where the final construction resulted in a terraced structure of over 730,000 m^3^ of soil [Fig. 7c;^6^].

At the moment, the available archaeological data for the Udine mound are few and do not allow to make solid hypotheses about the structures and activities that characterized its hilltop during Bronze Age. However, it is likely that, besides representing the power of the community through its monumental size, the mound would have been the site of some key buildings in the life of the settlement and of its related territorial system.

## Methods

Research methodologies are described in this section, with a detailed characterization for the most peculiar techniques, while for the conventional ones some more specific details are reported in the Supplementary information online.

### Historical and literary sources about the origin of the Udine Castle Hill

The legend about the construction of the mound of Udine by the legend of the construction of the Udine Castle Hill by Hun’s soldiers is deeply rooted in the local community and is reported in many different texts since Middle Age. The first written version of it back to the twelfth century, when it was recorded by the chronicler Godfrey of Viterbo and Otto, bishop of Freising^50^. We analyzed the main reference sources, focusing on the ones that proposed a slightly different explanation on the origin of the hill. In the Supplementary information online the original texts are reported with their translation in English from Latin and ancient Italian; moreover, a brief and objective comment is given about the interpretation of the origin.

### Archaeological excavations and geoarchaeological descriptions

We considered data collected from fresh archaeological excavations carried out at the Udine Castle Hill and the neighboring zone between 2020 and 2022. Beside the analysis of new researches, a review of all the pre-existing excavations in the area was carried out, considering the sites mentioned also in Visentini et al. [^23^; Figs. 2, 3 and 4 and Supplementary Figs. S1, S2)]. During recent fieldworks, the sedimentary characteristics, geometric and stratigraphic relationships, and geoarchaeological properties of the stratigraphic units were described. The dating of the different stratigraphic units was mainly based on the content of potsherds and other artifacts found within them.

### Typo-chronology of pottery finds and reference archeological periods

For the Bronze and Iron Age, the relative and absolute chronological phases and sub-phases presented in this work (see Supplementary Table S1) follow the chronological system developed by Cardarelli^17^ and, for Northern Adriatic, recently revised by Borgna et al.^21,22^ taking into account of the widely accepted Bronze Age seriation for Central Europe^51^.

### Stratigraphic cores

The deep stratigraphy of the study area was investigated through five mechanical cores drilled from the top of the Udine Castle Hill. Two of them were drilled in December 2020 (CAST-1 and CAST-2) up to a depth of 20 and 40 m, respectively, and three more cores were drilled in March–April 2022 (CAST-3, CAST-4 and CAST-5; Supplementary Fig. S6). Coring sites are indicated in Fig. 3 and Supplementary Table S2). A significant part of the research also considered the stratigraphic cores and sections previously described in the area of Udine city center, with a particular attention to those carried out in the Udine Castle Hill and in I Maggio Square.

### Radiocarbon dating

The geochronology of the deposits and archaeological structures was estimated through 10 AMS radiocarbon dates, that analyzed samples of wood and charcoals carried out at the CEDAD laboratory of the University of Lecce, the CIRCE laboratory at the University of Campania and the Ion Beam Lab of ETH in Zurich (Table 1).

### Paleobotanic analysis

Seven samples of sediment from the core CAST-2 were analyzed for microbotanical content at the following depths: 12.15, 18.65, 24.80–95, 30.95, 31.25, 31.40, 31.50, 31.70 and 31.85 m. The samples were prepared and analyzed at the Lab. of Palynology and Palaeoecology of CNR-IGAG in Milano according to standardized protocols (see Supplementary online). Diagrams were drawn using Tilia ver. 2.0.41 and Corel Draw X8 for further graphic elaborations.

### Topographic analysis and volume estimation of Udine mound and other sites

Topographic information about the study area was essentially derived from the analysis of digital terrain models (DTMs) produced from LiDAR data. For this purpose, we used LiDAR data gathered from the 2006–2007 survey carried out by the Civil Protection of the Regione Autonoma Friuli Venezia Giulia. The DTM of the area was freely downloaded from the official website of the Region (https://irdat.regione.fvg.it/CTRN/ricerca-cartografia/) and had a cell size of 1 m^2^ and a nominal vertical accuracy of ± 0.15 m. The elevation is referred to as above sea level (asl), based on the official Italian cartographic sea level, IGM1942.

Calculation of the current mound volume was derived from the altimetry of the natural ground surface detected around the Castle Hill and below it. Its identification was carried out in both archaeological excavations and stratigraphic cores, based on considerations derived from fresh information and data extracted from previous literature [e.g.^23,27^]. With this aim, we also reviewed unpublished material regarding the archaeological excavations carried out in the city center. This included sketches, photographs, and stratigraphic plans and sections) stored in the Raptor database, that is the archive of the Ministry of Culture—Soprintendenza Archeologia Belle Arti e Paesaggio of Friuli Venezia Giulia (https://www.raptor.beniculturali.it/; web access on November 2022), for the areas of Palazzo Dorta, Palazzo Contarini, Palazzo Mantica and Joppi Public Library (Figs. 2, 3 and 4 and Supplementary Figs. S1, S2).

The perimeter of the present mound was reconstructed through the analysis of topographic changes (from convex to flat) in the DTM, as indicated in Supplementary Fig. S9. At the base, the hill covers an area of about 44,500 m^2^. To obtain several estimations of the volume of sediment forming the present mound from the DTM, we subtracted the hypothetical horizontal surfaces corresponding to the base of the Castle Hill, which could be located at an elevation of 110 m, 111 m and 112 m asl. A similar calculation was made to assess the volume of the basin corresponding to I Maggio Square. In this case, the perimeter was drawn joining the points where, in the DTM, the topography changed from flat to concave.

To assess the volume of the prehistoric mound (i.e., without the historical additions detected along the rim of the hilltop), the geometry of the mound was simplified and different possible simulations were performed. On the basis of its present setting, we assumed that the mound had a round base and, as today, was symmetric also in ancient times. Nowadays, the circle inscribing the present base of the mound has a radius between 110 and 115 m. The stratigraphic data indicated that the prehistoric mound was similar to the present one. Nonetheless, we cautiously considered a radius of 100 m. In this, we took into account that the base of the Castle Hill could have expanded through millennia, although little evidence of colluvial material at the base has been documented to date.

Considering the data gathered in the 2021 excavation on the eastern sector of the hilltop, data from the CAST-5 core and information from the construction of the water tank in the mid-twentieth century CE (Fig. 6c), we hypothesized that the hilltop was also flat during prehistoric times, as the reworked deposits and historical debris in these areas are found within 2 m of depth from the present flat surface, which was located at about 141 m asl.

In assessing the volumes and dimensions of Silbury Hill (UK) and Cahokia Monks Mound (Illinois, United States), we reviewed relevant archaeological literature and compared information with the DTMs of both areas. For Silbury Hill, we processed the DTM of the area at 50 cm resolution, freely available from https://environment.data.gov.uk/DefraDataDownload/?Mode=survey. In this case, data were acquired in 2017 with a vertical accuracy of ± 15 cm root-mean-square error (RMSE) (https://data.gov.uk/dataset/977a4ca4-1759-4f26-baa7-b566bd7ca7bf/lidar-point-cloud).

For the Cahokia Monks Mound we produced the DTM of the area by downloading the original data that are freely available from https://clearinghouse.isgs.illinois.edu/data/elevation/illinois-height-modernization-ilhmp. The data were acquired in 2011 with a nominal pulse spacing (NPS) of 0.7 m. Raw LAS data were downloaded and processed to obtain a 50 cm resolution DTM.

### Total viewshed analysis

To evaluate the visibility from and to the Udine Castle Hill we performed the so-called total viewshed analysis. The analysis, performed in the software QGIS 3 through the Visibility Analysis plugin^52^, allows quantification of the extent to which each location within the area is visible from all other pixels of the grid according to a visibility index. This ranges from 0 to 1, where 1 is equal to 100% visibility and implies that a point can be seen from all of its neighbors^53–55^. The investigated area encompasses the piedmont strip, the upper, the lower and the coastal plain of Udine approximately between the Tagliamento and the Torre-Isonzo rivers. A 10-m DTM of the area, obtained from the re-sampling of both Italian and Slovenian LiDAR data at 50 cm resolution, was considered suitable for this purpose. Slovenian LiDAR data were downloaded at https://gis.arso.gov.si/evode/profile.aspx?id=atlas_voda_Lidar@Arso&culture=en-US and processed following the methodology developed in other areas of the Trieste Karst and described in previous publications (e.g.^56^. The radius was set at 10, 20 and 30 km; the sampling line of sight was at 8 and the observer height was at 160 cm. The analysis considered 41 sites including 8 fortified settlements, certainly coeval to the hill of Udine and 33 definite as Bronze Age tumuli documented in a recent survey^57^.

To test whether or not the position of sites was affected by a more extensive view across the landscape, a robust statistical approach, known as the Monte Carlo simulation, was adopted^58^. This test has been applied to visibility studies in archaeology since the late 1990s^59–61^. In our case, the Monte Carlo test was performed through the software RStudio and consisted of taking several random samples of points within the study area and comparing these with the archaeological samples of the considered sites (tumuli and castellieri). With this aim, in the first stage, we first set 'the convex hull' of all burial mounds as the window of analysis; second, we created a set of 99 samples of 41 random locations corresponding to the total number of considered sites, from which we extracted the mean of the visibility index of a 200 m buffer area. The results were then observed by visually comparing the empirical cumulative frequency distribution (ECDF) of the mean of the visibility index in each of the random sample with the sample of sites. Lastly, we obtained the p-value by ranking the mean of the slope of the 100 total samples in descending order and dividing the tumuli mean slope by the total number of means.

At a later stage, we considered the entire window of analysis to compare the visibility index for total viewshed maps at 30 km of 100 random points located on the top of the Castle Hill of Udine (observed sample) with the background population consisting of 99 samples of 100 points each, randomly located within the entire study area (the Friulian Plain).

### Toponyms and folklore traditions relating to Attila the Hun and other hills in NE Italy

Besides the Udine Castle Hill, the name of Attila the Hun is connected with several other places in NE Italy and reported in many tales, toponyms, or other folklore traditions. In particular, we selected places where a hill was present and analyzed the available sources of information. We also considered also the stories that are very well known by the local people, but are generally not described in any scientific paper, whereas they are reported only in the so-called “gray literature”.

## Supplementary Information

Supplementary Information 1.

Supplementary Information 2.

## Data Availability

All relevant data are available in the main text or in the Supplementary information.
