# Supplementary material for: The largest prehistoric mound in Europe is the Bronze-Age Hill of Udine (Italy) and legend linked its origin to Attila the Hun
Source: Sci Rep. 2023 May 31;13:8848. doi: 10.1038/s41598-023-35175-8 (PMC10232546; doi:10.1038/s41598-023-35175-8)
Supplement: Supplementary file 1 — Supplementary Information 1. [file 41598_2023_35175_MOESM1_ESM.pdf]

Stratigraphic core UD CAST-2 (46° 3'54.39"N - 13°14'10.41"E; 140.7 m a.s.l.)

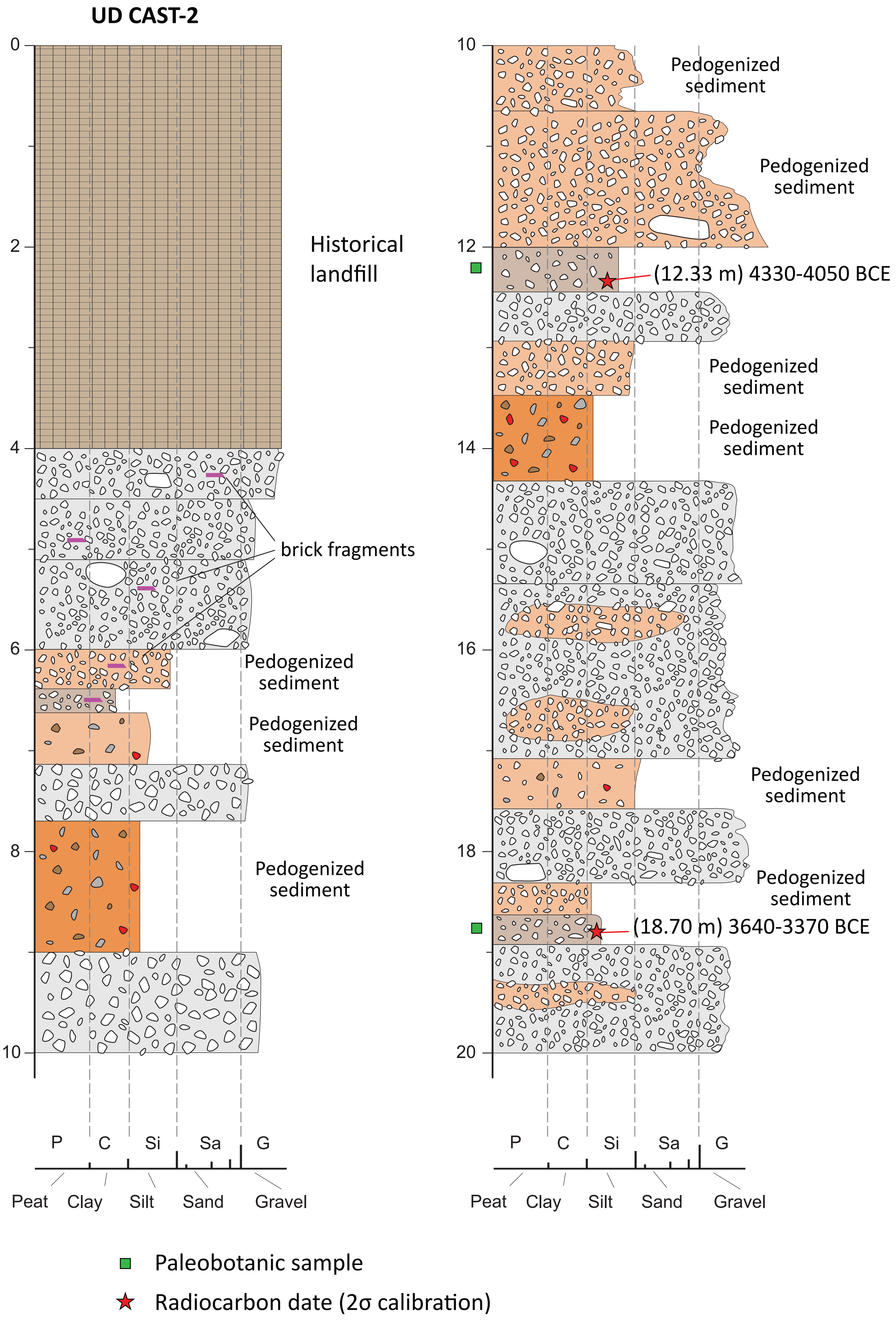

Stratigraphic core UD CAST-2 (46° 3'54.39"N - 13°14'10.41"E; 140.7 m a.s.l.)

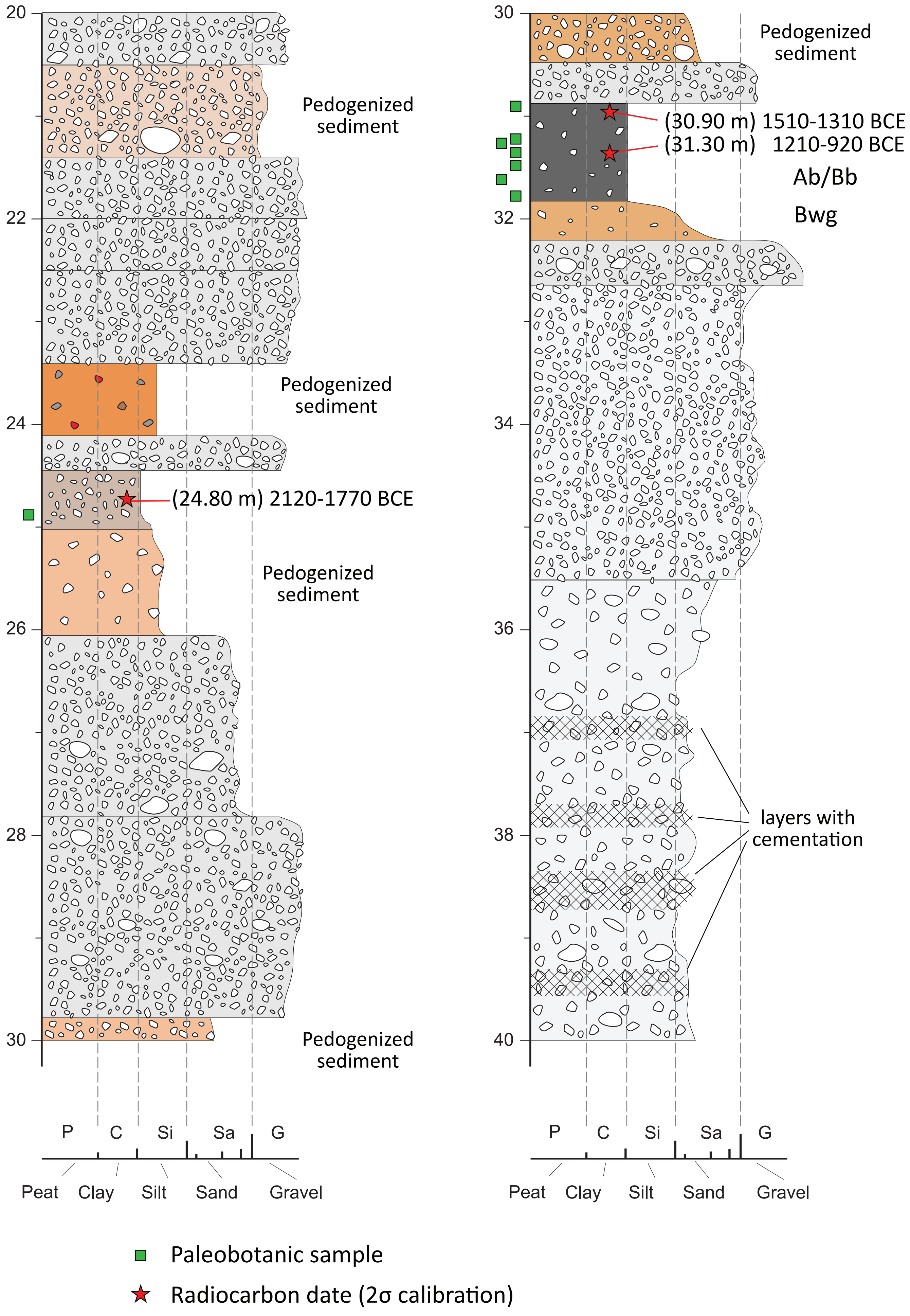

STRATIGRAPHIC CORE CAST-2

Udine Caste Hill (46° 3'54.39"N - 13°14'10.41"E; 140.7 m asl)

The core was drilled between December 3rd and 4th 2020. The cores are stored in the warehouse of the Museo Friulano di Storia Naturale in Udine. Colors of sediment were described according to Munsell Soil Color Charts (*1*); The content of carbonate calcium was assessed following the FAO-ISRIC soil description (*2*), according to the reaction to HCl (1 M), in a scale from 0 (no reaction) to 4 (bubbles >4 mm).

1. Munsell Color, *Munsell Soil Color Chart* (Baltimore, 1994).

2. FAO-ISRIC, *World reference base for soil resources 2006: a framework for international classification, correlation and communication*. (FAO, Roma, 2006).

STRATIGRAPHIC DESCRIPTION

0–4 m: Gravels and debris with variable matrix and containing common traces of anthropogenic activity (i.e., fragments of bricks of centimeter dimensions; plaster, mortar, lime and millimetric fragments of glass and charcoal). The interval corresponds to the outcrop described in the archaeological excavation in 2021 up to 3 m of depth and that was interpreted as a modern disturb consisting of reworked older deposits (cf. rubble). Below some specific layers of this interval are described.

0.65–0.85 m: clayey silts with organic component; color dark gray (2.5Y 4/1), HCl 4. Abundant remains of plaster and mortar with millimetric fragments of charcoal.

2.10–2.25 m: clayey silts with brick fragments; color very dark grayish brown (10YR 3/2); HCl 4.

2.70–2.80 m limestone cobble >10 cm, with traces of plaster.

3.40–3.50 m: clayey silts with brick fragments; color is dark brown (10YR 4–3/3).

3.70–3.80 m: level of backed red clay with traces of plaster/mortar; fragments of bricks and of overcooked bricks. The remains are compatible with parts of a structure used for fire-related activity.

4.00–4.45 m: medium-fine gravels with silts; pebbles have an average diameter of 1–2 cm, but rare cobbles have a maximum diameter of 4 cm. The matrix has a light gray color (2.5Y 7/2); (HCl 4). Some fragments of bricks at the top of this interval.

4.45–6.00 m: medium-fine gravels with silts; pebbles have an average diameter of 1–2 cm, whereas somw rare cobbles reach a maximum diameter of 4 cm. Color is light yellowish brown (10YR 6/4); (HCl 4). Some fragments of bricks at the top of this interval. The lower limit is sharp.

6.00–6.40 m: Clayey silts and silty clays with common pebbles of non-carbonatic lithologies and with very rare centimeter fragments of bricks. At 6.00–6.10 m presence of backed red clay. The color is variable from strong brown (7.5YR 5–4/6) to yellowish brown (10YR 5/4). HCl 0 in the reddish portions and HCl 1–2 in the yellowish ones. Traces of iron-manganese concentrations are present. The characteristics are comparable to the ones of pedogenized sediments. This interval is interpreted as the basal part of the landfill formed during recent and modern time through the reworking of older deposits including also archaeological structures and material.

6.40–6.60 m: slightly organic silt with abundant millimetric clasts and fine gravels and rare fragments of bricks; probable presence of plaster. Color dark gray (2.5Y 4/2); HCl 4. The layer was disturbed by coring operations.

6.60–7.20 m: over-consolidated silty clays with variable color, from brown (10YR 5/3) to strong brown (7.5YR 5/6); HCl 0–1. Common presence of pebbles, some with dimension up to 2 cm, with clear traces of

weathering and decarbonatation. Very rare occurrence of sub-centimeter brick fragments and probably "contaminated" from the higher layers during the coring operations. The lower limit is sharp. In general, but especially between 7.00 and 7.20 m, the layer consists of pedogenized sediment, with characteristics comparable to the ones displayed by the Bt2 and/or Bw horizons of the LGM surface in the surrounding plain. In particular, the reddish color of the matrix and the relative abundance of non-carbonate clasts, that are strongly weathered (e.g., Werfen formation, "Pietra verde of Cadore" formation), whereas the carbonatic ones are very limited in number and show clear traces of dissolution.

7.20–7.70 m: fine gravels in clayey silt matrix; color pale brown (10YR 6/3); HCl 4.

7.70–9.00 m: clayey silts, silty clays and gravels, color strong brown (7.5YR 5/4); HCl 3–4. Cobbles of 4–5 cm from rare to common. Sharp lower limit. Sediment is pedogenized.

9.00–10.00 m: medium-fine gravels with silts; color light gray (10YR 7/2); HCl 4. Average diameter of pebbles is 1–-2 cm and rarely up to max 4 cm. Sharp lower limit.

10.00–10.60 m: clayey silts and silty clays that form the matrix of medium-fine gravels, with pebbles of max 2–3 cm of diameter; color dark brown (7.5YR 4/4); HCl 3–4. The characteristics are compatible with pedogenized sediments.

10.60–12.00 m: clayey silts and silty clays and medium-fine gravels with pebbles of max 2–3 cm of diameter. Presence of a cobble >10 cm at the bottom. Color dark brown (7.5YR 5/3) that is lighter than the overlying layer; HCl 4.

12.00–12.40 m: clayey silts with weak organic component; color brown to yellowish brown (10YR 5–4/3); HCl 0. A millimetric charcoal fragment at 12.33 m. The gravel clasts are almost all carbonatic. This layer probably corresponds to sediment reworked from an A horizon of a humid soil or to an in-situ weakly developed soil.

12.40–12.90 m: fine gravels with silts; color dark brown (7.5YR 5/3); HCl 4.

12.90–13.40 m: silty clays with common pebbles of 3–4 cm; color brown to strong brown (7.5YR 5/4–6); HCl 4. These characteristics are compatible with slightly pedogenized sediments.

13.40–14.30 m: strongly consolidated silty clays with gravels, with presence of rare iron-manganese nodules; color brown (7.5YR 4/3); HCl 0. Pebbles are generally strongly weathered. The characteristics are compatible with pedogenized sediments.

14.30–17.10 m: clayey silts with pebbles with an average diameter of 1.5–2 cm, but rare clasts >10 cm were present at 14.60 m and 14.80 m. The color is darker in rare sectors (i.e., dark brown 7.5YR 5/3), whereas is generally light yellowish brown (10YR 6/4); HCl 4. The characteristics are compatible with the occurrence of small lenses of pedogenized sediments. At 15.50 m a wooden remain was found, probably part of a seed.

17.10–17.60 m: clayey silts with fine gravels and rare pebbles; color light yellowish brown (10YR 6/4); HCl 0–1. At 17.40 m a centimeter fragment of strongly weathered non-carbonatic rock. The characteristics are compatible with pedogenized sediments.

17.60–18.40 m: clayey silts and medium-fine gravels; color yellowish brown (10YR 5/4); HCl 4. At 18.10 m presence of a cobble of limestone >10 cm.

18.40–18.60 m: silty clays with medium-fine gravels; light brown (7.5 YR 6/4); HCl 1–2. The characteristics are compatible with pedogenized sediments.

18.60–18.90 m: clayey silts with weak organic component; color dark to very dark grayish brown (10YR 4–3/2); HCl 0–1. Common pebbles with average diameter of 3 cm and presence of iron-manganese mottling with evidenced millimetric lamination. Rare millimetric fragments of charcoal. This layer probably corresponds to sediment reworked from an A horizon of a humid soil or to an in-situ weakly developed soil.

18.90–20.00 m: clayey silts and silty clays with medium-fine gravels; variable color up to dark brown (7.5YR 4/3); HCl 3. At 19.60 m a cobble >10 cm. The characteristics of the layer are compatible with the occurrence of small lenses of pedogenized sediments within a layer mainly composed of unweathered material.

20.00–20.50 m: clean medium gravels with some cobbles >10 cm at the top and the bottom of the layer. Probably the matrix was washed away by the drilling operation.

20.50–21.40 m: clayey silts and silty clays with medium-fine gravels; at 21.30–21.40 m a cobble >10 cm is present. Variable color with maximum tone yellowish brown (10YR 5/4); HCl 0–1 at the top and increasing content of carbonate towards the bottom.

21.40–22.00 m: medium to fine gravels with silts. Common cobbles of 3–5 cm; variable color, up to yellowish brown (10YR 5/4). The core is quite reworked by the drilling operation.

22.00–22.50 m: fine gravels with silty clays; color grayish brown (10YR 6/3); HCl 4. The interval was strongly reworked by the drilling operation.

22.50–23.40 m: clayey silts with medium-fine gravels; very rare cobbles up to 5 cm. Variable color from brown (7.5YR 5/3) to yellowish gray (10Y 6/3); HCl 4. The characteristics are compatible with pedogenized sediments.

23.40–24.10 m: silty clays, well consolidated, with fine to medium gravels; between 23.95–24.10 m a cobble >10 cm. Polyhedral strong weathered pebbles are common. Color brown (7.5YR 4/3); HCl 0. The characteristics are compatible with pedogenized sediments.

24.10–24.40 m: clayey silts with medium-fine gravels.

24.40–25.00 m: clayey silts and silty clays, with low organic content and rare millimeter fragments of charcoal. Presence of rare clast and pebbles. Variable color, generally dark grayish brown (10YR4/2), but in the basal portion there are some dark brown lenses (7.5YR 4/3); HCl 0. The characteristics are compatible with pedogenized sediments, including part of an A horizon. It is also possible that the layer corresponds to part of a humid soil or to an in-situ weakly developed soil.

25.00–25.20 m: clayey silts with fine-medium gravels. Color light brown (7.5 YR 6/4); HCl 4.

25.20–26.05 m: silty clay with presence of fine gravels and rare pebbles <2 cm and isolated cobbles; color dark brown (7.5YR 4/3) at the bottom that becomes lighter in the upper part (10YR 6/4); HCl 0. The characteristics are compatible with pedogenized sediments.

26.05–27.80 m: clayey silts with fine gravels, with rare pebbles up to 2–4 cm. Color light brownish gray (10YR 6/2). The whole interval has been reworked by the coring operations.

27.80–29.80 m: clayey silts with fine gravels, with common pebbles up to 4 cm. Color yellowish brown (10YR 5/4). The whole interval has been reworked by the coring operations.

29.80–30.60 m: consolidated silty clays with fine gravels; color light brown (7.5 YR 6/4). The characteristics are compatible with pedogenized sediments.

30.60–30.90 m: clayey silts with fine gravels; color dark yellowish brown (10YR 4–3/4); HCl 4.

30.90–31.80 m: clayey silts with organic material dispersed in the matrix; the color is progressively darker up to 31.30–31.60 m. Rich in very fine gravel at the top up to 31.20 m with charcoal at the top. Presence of faint traces of millimetric charcoals up to 31.20 m. From 31.20 to 31.70 m presence of clasts packed in the matrix but quite isolated, generally from pluri-millimetric to 15 mm; however also rare isolated centimeter clasts. At the top 10YR 3/4-3, HCl 2–3. At 31.40 m very dark gray 10YR 3/1, HCl 1. At 31.60 very dark gray to very dark grayish brown 10YR 3/1–2, HCl 0–1. Throughout the layer there are rarely millimetric

charcoals or plant macroremains that are weathered with different degrees. The layer corresponds to the A/B horizon of a soil, likely reworked or influenced by the ancient anthropogenic activity.

31.80–32.20 m: clayey silts with faint organic component that decrease downwards; color dark brown (10YR 4/3); HCl 0–1. Presence of iron-manganese millimetric laminae at the base that evidence the mottling. Lower limit is sharp. The layer corresponds to the Bw horizon of a soil.

32.20–32.60 m: medium gravels with silts and presence of rare pebbles up to 4 cm; color light gray (10YR 7/2); HCl 4.

32.60–35.70 m: medium-fine gravels in a silty-sandy matrix with some rare cobbles up to 5 cm. Color light gray (10YR 7/2); HCl 4. The layer is strongly different the layers above and below it. The lower limit is sharp.

35.70–40.00 m: sandy silt with fine gravels and pebbles <2 cm from rare to common; very rare isolated pebbles up to 6 cm. Color light gray (10YR 7/2); HCl 4. Rare occurrence of slightly cemented horizons that have a decimeter thickness. Lower limit unknown.
