# Supplementary material for: The largest prehistoric mound in Europe is the Bronze-Age Hill of Udine (Italy) and legend linked its origin to Attila the Hun
Source: Sci Rep. 2023 May 31;13:8848. doi: 10.1038/s41598-023-35175-8 (PMC10232546; doi:10.1038/s41598-023-35175-8)
Supplement: Supplementary file 2 — Supplementary Information 2. [file 41598_2023_35175_MOESM2_ESM.pdf]

Supplementary Information for

The largest prehistoric mound in Europe is the Bronze-Age Hill of Udine (Italy) and legend linked its origin to Attila the Hun

Authors: A. Fontana[1]*, G. Vinci[1,2], L. Ronchi[1], A. Mocchiutti[3], G. Muscio[4], P. Visentini[4], M. Bassetti[5], M.D. Novellino[1,6], F. Badino[1,6], G. Musina[7], S. Bonomi[7]

Affiliations:
[1]Department of Geosciences, University of Padova; Padova, Italy.
[1,2]Department of History and Cultural Heritage, University of Siena; Siena, Italy
[3]Geomok Srl; Udine, Italy.
[4]Museo Friulano di Storia Naturale, Comune di Udine; Udine, Italy.
[5]Cora Società archeologica srl; Trento, Italy.
[6]CNR-IGAG, Laboratory of Palinology and Paleoecology, Milan.
[7]Soprintendenza Archeologia belle arti e paesaggio del Friuli Venezia Giulia; Trieste, Italy.

*Corresponding author: alessandro.fontana@unipd.it

This PDF file includes:

Materials and Methods
Supplementary Text
Figures S1 to S14
Tables S1 to S2

Other Supplementary information for this manuscript include the following:

Data S1 - Stratigraphic log and description of CAST-2 core

Materials and Methods

Historical and literary sources about the origin of the Udine Castle Hill

The legend about the construction of the mound of Udine by the Huns of Attila is deeply rooted in the local community and is reported in many different texts. We started from the work of Achille Tellini (1900), the geologist that first described the geomorphology of the area of Udine and compared the landforms with the most ancient written sources. In particular, we re-analyzed the original texts written in the 12th century by the bishop of Freising, Otto [1158, (*1*)] and Godfrey of Viterbo [1198, (*2*)]. Moreover, we also considered other sources produced between 16th and 19th centuries, where the legend of Attila is described, but also where other explanations for the origins of the Udine Castle Hill are discussed. In particular, in the supplementary text, we report the original texts in Latin or in ancient Italian, with their English translation and a brief comment on the content. The written sources are: Marin Sanudo Il Giovane (*3*), Erasmus of Valvasone (*4*), Giambattista Albrizzi (*5*), Paolo Fistualario and Jacopo Valvasone (*6*).

Archaeological excavations and geoarchaeological descriptions

In this research, we considered fresh data collected from archaeological excavations carried out at the Udine Castle Hill and the neighboring zone between 2020 and 2022. Beside the analysis of new researches, a review of all the pre-existing excavations in the area was carried out, considering the sites mentioned also in Visentini et al. [(*7*); Figs. 4, Supplementary Figs. S1, S2).

Between October and December 2021, in the framework of the project for building the elevator reaching the top of the Udine Castle Hill from its eastern side, the Comune of Udine funded an archaeological excavation directed by the Ministry of Cultural Heritage (Soprintendenza Archeologica Belle Arti e Paesaggio del Friuli Venezia Giulia; Figs. 4-5 and S3). The investigation consisted of an 8 x 12 m test pit that reached 3 m in depth from the present surface of the esplanade characterizing the hilltop. It was opened in the eastern sector of the hill, 8 m south of the building named "Casa della Contadinanza" (Figs. 4, Supplementary Figs. S1, S2).

Between October 2020 and January 2021 an archaeological excavation was carried out in the basement of Palazzo Dorta, at the south-western foot of the Udine Castle Hill, near Libertà Square (Figs 4, S2 and S4A). Between April and June 2022, an archaeological excavation was carried out near Palazzo Contarini, close to Manin Street, and at a short distance from Palazzo Mantica (Figs. 4, S2, and S4B).

In the different sites, during the fieldwork, the depositional sequence was described distinguishing stratigraphic units (US) that were identified in the pit sections and logged by hand drawing and photogrammetric survey at centimetric accuracy and precision. The sedimentary characteristics, geometric and stratigraphic relationships, and geoarchaeological properties of the stratigraphic units were described. In particular, as well as the archaeological structures and artifacts, the description of each stratigraphic unit covered the texture, color, consistency, and reaction to HCl (1 M) for assessing the quantity of carbonates. The description followed FAO-ISRIC soil description rules (*8*). Colors were defined according to the Munsell Soil Color Chart (*9*).

The dating of the different stratigraphic units was mainly based on the content of potsherds and other artifacts found within them.

As regards the area of the hill, among the previous archaeological excavations, particular emphasis was given to the eastern edge of the hilltop, investigated in 1986–1987, near the building named "Casa della Confraternita" (Fig. 5B).

Typo-chronology of pottery finds and reference archeological periods

For the Bronze and Iron Age, the relative and absolute chronological phases and sub-phases presented in this work (see Supplementary Tab. S1) follow the chronological system developed by Cardarelli (*10*) and, for Northern Adriatic, recently revised by Borgna et al. (*11*) taking into account of the widely accepted Bronze Age seriation for Central Europe (*12*). As regards the dating of finds, the Bronze Age (2200–950 BCE) is the first archaeological period in Northern Italy that allows a well-defined and robust differentiation of chrono-typological phases, with an accuracy of 20–100 years, based on assemblages of pottery and metal artefacts, as well as on dendrochronology and radiocarbon dating (*13*).

In this research, particular attention was devoted to the pottery fragments found in the infill of the structure named "Fossa Bronzo" (Bronze-Age pit; Fig. S5) and investigated in 1987 along the eastern edge of the hilltop, near the building called Casa della Confraternita (*14*). The typo-chronological analysis of the pottery assemblage was carried out by Giovanni Tasca (*15*), based on relevant comparisons with significant Bronze Age contexts from the northern Adriatic area. Together with the rest of the archaeological finds collected in the archaeological excavation of 1987, the shards are stored in the Musei Civici in the Castle of Udine.

Stratigraphic cores

The research investigated the subsoil of the core thanks to five new stratigraphic cores carried out between 2020 and 2022. In December 2020, two mechanical cores CAST-1 and CAST-2 were drilled from the top of the Udine Castle Hill up to a depth of 20 and 40 m, respectively (for location see Fig. S2 and Table S2). After their study, three other cores were drilled in March-April 2022 (i.e., CAST-3, CAST-4 and CAST-5; Fig. S8). All the cores were collected using a mechanical probe mounted on a truck. The drilling operations were carried out with a simple single corer of 101 mm in diameter and a casing of 127 mm for S1 and S3, while for the first 25 m of the other cores, the corer had a diameter of 127 and casing of 152 mm. The coring operations were performed in dry conditions and limited use of water to avoid the fluidification of sediments, with the aim of limiting the disturbance induced by the drilling procedures. The collected cores were enveloped in nylon film for every single meter of stratigraphy and stored in plastic boxes immediately after their recovery. The cores are stored in the warehouse of the Museo Friulano di Storia Naturale in Udine. The locations of the cores and their characteristics are visible in Figs. 2A, S1, S6 and Table S2.

A preliminary description of each core was undertaken in the field during the coring operations, together with their brief recording and photographic documentation. The cores were later described in detail at the Museo Friulano di Storia Naturale in Udine, where they were opened. The rare portions characterized by fine deposits (i.e., silts, clays and sandy silts) were cut in two halves using a steel harmonic wire of 0.5 mm in diameter, while the portions where coarse materials were dominant (i.e., gravels) were opened with putty knives. The texture of sediments and matrix of the cores were described using empirical manipulation, according to the USDA classification (*8*). The color of the sediments was defined in humid conditions using the

Munsell Soil Color Chart (*9*), and the carbonate content was assessed by observing the reaction with HCl (1 M). Where the occurrence of traces of soil-forming processes were identified in the sediments, the description of pedogenetic characteristics and the designation of soil horizons followed the FAO-ISRIC classification (*8*). The intervals where organic content was detected in the matrix because of its dark color, were carefully inspected, looking for roots, wood and charcoal fragments. Samples of charcoals and woods were taken for further paleobotanic and geochronological investigations. If present, sedimentary structures (e.g., layering, alternations, laminations) were described. Some portions of the gravelly sediments were washed and cleaned from the matrix in order to detect whether glacial striations were present on the surface of any pebbles that could perhaps be related to glacial transport. The portions of the cores where archaeological artifacts or their fragments were present were carefully inspected and the prehistoric archaeological features have been sampled in plastic bags.

For our research the reference stratigraphy for the Udine Castle Hill was based on the CAST-2 core, and its detailed stratigraphic description and log are reported in the separate file Data S1: Stratigraphic description and log of core CAST-2. The logs of the other new cores carried out within our study are reported in Fig. S8.

A significant part of the research also considered the stratigraphic cores and sections previously described in the area of Udine city center, with a particular attention to those carried out in the Udine Castle Hill and in I Maggio Square. Major sources of information were the data reported in Bernardis and Zorzi (*16–17*), Comel (*18–23*), Feruglio (*24–25*), Lorenzi (*26*), Martinis (*27*), Paronuzzi (*28*), Zorzi (*29*) and the review published by Zanferrari et al. (*30*). In particular, Bernardis and Zorzi (*16*) described the cores drilled in 1976 during restoration works on the Castle of Udine after the earthquake that occurred that year and, on the basis of those data, they already recognized that a significant portion of the hill corresponds to artificial landfill. However, they clearly subscribed to the idea that the Udine Castle Hill had mainly originated because of tectonic thrust activity, which would have uplifted the pre-Quaternary bedrock to the surface.

Radiocarbon dating

The geochronology of the deposits and archaeological structures was estimated through several AMS radiocarbon dates, that analyzed samples of wood and charcoals (Table 1). In particular, five dates were carried out on samples from the CAST-2 core, which was drilled from the eastern edge of the hilltop, inside the area investigated by the archaeological excavation in 2021, and it was the first core that passed through the whole sedimentary body of the mound. Moreover, the CAST-2 core was quite close to the wartime tunnel A, where a wooden artifact was found in 1943, since when it has been preserved in the Musei Civici of Udine (archive number 130). After a visual inspection by paleobotanist Mauro Rottoli, a millimetric fragment of this archaeological find was selected and sent to the laboratory for radiocarbon dating.

The selected samples were dated using the AMS technique at the CEDAD laboratory of the University of Lecce, the CIRCE laboratory at the University of Campania and the Ion Beam Lab of ETH in Zurich. After the collection, samples were sealed in aluminum sheets and in plastic bags and sent to the laboratory.

The sample from stratigraphic unit 107 was collected in December 2021 in the archaeological excavation on the eastern sector of the hilltop. It consisted of a centimetric fragment of charcoal that was found within the silty gravels, representing one of the lenses that formed the top of the

anthropogenic mound. The sample was collected 40 cm below the erosive surface, marking the boundary with the ground of the early medieval age (Fig. S3B).

The results of the radiocarbon dates are reported along the main text in Table 1. After their radiocarbon analysis, the results were calibrated with the software Calib 8.2 (*31*) using the IntCal20 curve (*32*). The calibrated values were rounded out to 10 years.

Paleobotanic analysis

From the core CAST-2 seven samples of sediment were collected and analyzed for microbotanical content at the following depths: 12.15, 18.65, 24.80–95, 30.95, 31.25, 31.40, 31.50, 31.70 and 31.85 m. The samples were prepared using standard methods (including HF and acetolysis) after adding Lycopodium tablets for pollen concentration and influx estimations (*33*) at the Lab. of Palynology and Palaeoecology of CNR-IGAG in Milano. Pollen identification was carried out at ×400, ×630 and ×1000 magnifications under a light microscope, following Beug (*34*), Punt et al. (*35*), Reille (*36–37*), and the pollen reference collection of the CNR lab in Milan. The pollen sum used for % calculations included trees, shrubs, and all upland herbs, and for each sample a minimum count of 600 grains was reached, excepted for the deepest one at 31.80 m. Aquatics, spores and any other microbiological particles other than pollen were analyzed but excluded from the pollen sum. Poaceae pollen types were classified based on grain diameter (D) and annulus diameter (d): wild grasses (D<47 μm + d<11 μm), and Poaceae including wild and cereal species (D>47 μm + d<11 μm). Poaceae with D>47 μm and d>11 μm are referred to cereals [i.e., cultivated *Avena* and *Triticum* species, Cerealia type sensu (*38*)]. *Plantago lanceolata*, *Orlaya grandiflora* type and cereals were grouped into synanthropic and cultivated taxa. Besides, *Sordaria* type and Sordariaceae spores were also taken into account as indicators of dung-related fungi.

Pollen-slide microcharcoal particles were recognized under a light microscope at ×400. Black, completely opaque and angular fragments (*39*) were identified as charcoal within the size classes 10–62, 62–125, 125–250 μm length. Diagrams were drawn using Tilia ver. 2.0.41 (*40*) and Corel Draw X8 for further graphic elaborations.

Data and Results

Literary and historical sources about Attila the Hun and the Hill of Udine

The origin of the Hill of Udine is described in several historical sources that present some differences, but all of them refer to the artificial construction of the mound by Attila the Hun and, some to the hypothesis that it was built by the Romans (often Julius Cesar). The principal ancient and historical written sources are reported below.

Otto, bishop of Freising [Ottonis Episcopi Frisingensis Chronica Sive Historia De Duabus Civitatibus, ed. Adolf Hofmeister, Hannover, Hahnsche Buchhandlung, 1912. Monumenta Germaniae Historica. Scriptores. Scriptores rerum Germanicarum in usum scholarum separatim, vol. 45; Liber IV, chapter 27; (*1*)]. The author (1111–1158) describes the Hill of Udine in the first half of 12th Century and, besides the story thatthe mound was built by the Huns, he also reports the alternative hypothesis that the mound was constructed by the Romans of Julius Caesar.

Original text: "*Tanta autem in obsidione huius Urbis (Aquilejae) et mora, et multitudo fuit, ut mirae magnitudinis montem Utinum dicunt, quem ipse vidi, in modum aggeris ab exercitu comportatum incolae usque hodie affirment. Alii tamen a Iulio Cesare eundem montem factum tradunt.*"

Translation: "The siege of this city (Aquileia) was so prolonged, and such was the multitude involved, that they say that the grandiose hill of Udine, which I myself have seen, was collected in an earthwork by his (Attila's) army, as the inhabitants of that city still affirm. Others, however, say that this mountain was built by Julius Caesar."

Godfrey of Viterbo [Godefridi Vieterbensi, Pantheon Seu Memoria Seculorum, par. XVI, tomo VII, Rer. Ital. col. 376], in the version reprinted by the Venetian printer Ludovico Antonio Muratori in 1725 [Ex typographia Societatis Palatinae in Regia Curia; (*2*)]. The author (1138–1197), in the 12th century described the origin of the hill as a mound built by the Huns of Attila but, in contrast to the current version of the legend, the soldiers used their shields and not the helmets for transporting the earth. A most important point is that the author presented the alternative hypothesis that the mound had been constructed by Julius Caesar.

Original text: "*Exercitus Atilae ibi tantus fuit, quod suis clypeis aggerem ad similitudinem rotundi Montis, ob futuram memoriam Atilae comportavit. Alii vero dicunt eum per Cesarem Julium fuisse congestum. Ego Gotfridus montem illum vidi meis temporibus bene munitum et inhabitatum.*"

Translation: "Attila's army was so large there that it piled up an earthwork in the shape of a round mountain with its shields to celebrate the future memory of Attila. Others, however, say that it was piled up by Julius Caesar. I, Gottfried, saw that mountain, which in my days was well fortified and inhabited."

Marin Sanudo il Giovane [Descriptione de la Patria de Friuli, Descriptio Terre Utini; (*3*)]. The Venetian historian Marin Sanudo il Giovane (1466–1536), in his book written in 1502–1503, described the region of Friuli and dedicated a paragraph to the city of Udine in which he reported the legend of the origin and described the castle that was present on the hilltop at that time.

Original text: "*Divulgasi che non da natura ma per il Tyranno Atila il quale in quel tempo havea zurato lo excidio de italia la dove horra e udene fu manualmente facto un monte de terra*

sul alteza del quale hora per una bella via copertavoltata su collone 52 se li assende per gradi 125 et puol volzere dal pie zercha miglio mezo et in cima cinto de muro in forma de castello e fabricato con una Torre due chiesie et due cisterne uno assai bel palazo che domina tutto el piano col suo prospecto."

Translation: "It is said that not by nature but by the tyrant Attila, who at that time had sworn the massacre of Italy, an earthen mountain was built by hand where Udine is now, and today you can climb to the top of it along a beautiful street, covered by a portico with 52 columns and formed by 125 steps and about half a mile long and at the top there is a beautiful mansion , surrounded by a wall like a castle and formed by a tower, two churches and two cisterns and which has a view over the whole plain."

Erasmo of Valvasone [Poema della Caccia; (*4*)]. The author (Valvasone, 1523–Mantova, 1593), in his poem, published in 1591, wrote about the Hill of Udine and, as others, he mentioned the fact that also the name "Udine" derives from the ancient "Utinum", from "Unnium" related to the Huns, that in Italian are called "Unni".

Original text: *"Nel mezzo sede la città ch'eresse / Attila, e gl'Unni, onde il suo nome ottenne / Nobil città, che ad Aquileia successe*".

Translation: "In the middle there is the city built by Attila and the Huns, from whom the noble city derived its name and that succeeded Aquileia."

Giambattista Albrizzi [La Patria Del Friuli; Descritta Ed Illustrata Colla Storia E Monumenti Di Udine Sua Capitale E delle altre Città e Luoghi Della Provincia, Stamperia Albrizzi, Venezia, 1753; B3–B4; (*5*)]. This Venetian historian, in his book published in 1753, described in detail the city of Udine and discussed the origin of the hill. In a significant passage the author questioned the story that the hill was built by the Romans. He argued that some of his contemporary scholars are supported the idea on the basis of an ancient stone inscription found during the restoration of the castle, but he is not convinced of the authenticity of the stone. Moreover, he commented the poem of Erasmus of Valvasone, who wrote that the core of the hill was made of solid rock (vivo sasso).

Original text. "*Molte ciance intorno all'origine di questo colle, e al suo antico castello si fanno, e molte false tradizioni si spacciano. Chi pretende essere stata opera degli antichi Romani, e si dà ad intendere esservi stata scoperta fra le rovine dell'antica, nella erezione della nuova fabbrica, un'antica pietra colla sua iscrizione riportata dai due Palladj, le Sigle della quale vengono spiegate da Flaminio Flaminio, che per avventura potrebbe essere stato l'Autore; quando non sia stato il P. Vecchia da Udine Monaco Camaldolense, come sospetta Monsignor del Torre. Altri suppongono questa elevazione di terra essere artefatta, e che Attila nella irruzione che fece in Italia, e nel tempo dell'assedio di Aquileia l'avesse da' suoi unni fatta formare per quindi vagheggiare le fiamme della incendiata Aquileja, onde Unnium dagli Unni avesse la città a chiamarsi. Così Sabellico nella sua Storia dell'antichità di Aquileia, ed in un suo Poema sopra la origine della stessa città di Udine, ov'ei fu Precettore, cerca di dar ad intendere, seguito poi da molti altri Storici del paese, ci è piaciuto quello ingegnoso ritrovato. Sul qual fondamento appoggiato Erasmo di Valvasone nel suo Poema della Caccia così cantò:*

Nel mezzo sede la città ch'eresse / Attila, e gl'Unni, onde il suo nome ottenne / Nobil città, che ad Aquileia successe. Verisimil cosa ella si è, che questo colle di vivo sasso, e di terra dalla natura formato, ed innalzato in mezzo a questa amena pianura, abbia dato ne' tempi antichi occasione di fabbricarvi sopra un Castello con case al medesimo annesse…."

Translation: "There are many stories about the origin of this hill and its ancient castle, and many false traditions are passed off. Some claim it was built by the ancient Romans and maintain that, during the construction of the new building, an ancient inscribed stone was discovered in the ruins of the previous building, and that the inscription was reported by the two Palladios [artists working in Udine], the letters on which are explained by Flaminio Flaminio, who was perhaps their Author; unless it was Padre Vecchia from Udine, a Camaldolian monk, as suspected by Monsignor del Torre. Others suppose this elevation of land to be artificial, and that during his attack on Italy, and at the time as his siege of Aquileia, Attila had it built by his Huns in order to watch the flames of Aquileia as it burned, so that the city's name Unnium is derived from the Huns. So Sabellico maintains in his History of the antiquities of Aquileia and in one of his Poems on the origin of the same city of Udine, where he was Preceptor, and is followed by many other historians of the region, and I like this explanation. Following this assertion, Erasmo of Valvasone wrote as follows in his Poem on hunting: *Nel mezzo sede la città ch'eresse / Attila, e gl'Unni, onde il suo nome ottenne / Nobil città, che ad Aquileia successe. Verisimil cosa ella si è, che questo colle di vivo sasso, e di terra dalla natura formato, ed innalzato in mezzo a questa amena pianura, abbia dato ne' tempi antichi occasione di fabbricarvi sopra un Castello con case al medesimo annesse…."[this poem is translated in the previous paragraph about Erasmo of Valvasone].*

<u>Paolo Fistualario and Jacopo Valvasone</u> [Monografie Friulane offerte a Monsignore Zaccaria Bricito, arcivescovo di Udine ece., Udine Vendrame, 1847; (*6*)], wrote that the most authentic ancient monument in Udine is the hill, that corresponds to a mound built in ancient times quarrying the gravels and earth from the nearby area named Giardino (now I Maggio square), where the existing depression documents this activity. Moreover, they describe that in all the outcrops and excavations in the hill, even at its core, no traces of rock have been found, but simply gravel and earth. The authors clearly write that popular tradition attributes the construction of the hill to the Romans, as they were the champions in building large constructions, but the age is unknown.

Original text, pag. 12–14: "*Quindi è che monumento più autentico e più sicuro dell'antichità di Udine io non saprei immaginare dell'istesso colle, ove gettate furono ne' tempi addietro le prime sue fondamenta. Antichissima tradizione è, come poco fa accennammo, che esso colle non dalla natura, ma alla mano degli uomini e dall'arte fosse stato, sin dai secoli che noi chiamiamo romani, in mezzo al piano innalzato. Vero è però che una tale tradizione di nuin conto fu riputata da un degno e dotto nostro scrittore, che considerò anzi il nostro colle di vivo sasso e di terra dalla natura formato, ed innalzato in mezzo a questa amena pianura, né io sarei punto lontano dall'unirmi seco lui in opinione, quando la cosa unicamente dalla tradizione dovesse dipendere. Ma dacchè ognun può vedere, che alla tradizione corrisponde a meraviglia la profondità del pian ben vasto, che a piè del colle si stende, e che giardino si dinomina, sarà permesso a me il dipartirmi da un tal sentimento, senza perdere né anche una minima particella di quella stima, nella quale io tengo meritatamente l'eruditissimo autore.*

Dissi che alla tradizione corrisponde mirabilmente la profondità del piano, o sia valle, che osservasi a piè del nostro colle, e che giardino appellasi comunemente, perché fattone a di nostri a bella prova esperimento, si è ritrovato mancare alla valle stessa, in paragone dell'ampia restante pianura ov'è situata, molto più di terra che non richederebbesi ora per formar di pianta l'altezza tutta del colle. Da una tale mancanza straordinaria di terra che forma la detta valle, contro la natura e perfezione del piano ove sprofondasi, e dalla rotondità in appresso e rarità

del colle che in confine della medesima s'innalza, senza esempio in vicinanza, per lo spazio di molte e molte miglia, di eminenza alcuna benchè minima di terra, chi sarà mai che non conchiuda, il nostro colle fabbricato essere dall'arte e non dalla natura, e formato colla terra stessissima, che alla radice di esso manifestamente trovasi a mancare? Né mi si dica che l'interno di questo colle sia composto in parte di vivo sasso, perché ciò non si verifica punto, come si è veduto ultimamente nello scavamento seguito nel cuore stesso del colle, in occasione di rifare le antiche e profonde sue cisterne, ove non si è incontrato alle pareti delle medesime neppure indizio di vivo sasso o di macigno, ma bensì tutta terra ghiaiosa; ciò che istessamente fu osservato altre volte, e può osservarsi in tutto il colle da capo a piede, a riserva di quel pezzo tenero e fragil tufo solito generarsi col tempo anche altrove in vicinanza alla superficie degli edifizj e delle muraglie. Bel monumento per vero dire è questo, durevole e non soggetto ad abbaglio o alterazione, o ad esserci stato qui portato d'altronde, e di antichità tanto più grande e più lontana, quanto che ella si toglie affatto al nostro sguardo, e se ne sta tuttavia immersa e giacente nell'oscurità dei secoli più remoti; onde il volgo attonito in processo di tempo non seppe attribuire ad altri la grande impresa, che ai Campioni più rinomati dell'antichità; indicando con ciò manifestamente alla posterità la somma antichità del luogo, quale poi la riconobbero anche i due celebri ristoratori della Geografia antica, Cluverio e Cellario, e con esso loro il mentovano chiarissimo sig. Marchese Maffei."

Translation: "Thus, I would not know how to imagine a more authentic and surer testimony of the antiquity of Udine than the hill itself, where the first foundations of the city were laid in ancient times. It is a very ancient tradition, as we mentioned a little while ago, that this hill was raised in the middle of the plain, not by nature, but by the hand and skill of men, from the Roman period. It is true, however, that this tradition was considered worthless by an illustrious and learned writer of ours [Erasmus of Valvasone], who conversely considered our hill to be solid rock and earth and formed by nature, and raised in the middle of this pleasant plain, nor would I be far away from joining him with this opinion, if the thing only depended on tradition. But since everyone can see that the tradition fits perfectly with the vast basin, which stretches out at the foot of the hill, and that is called the Garden [I Maggio Square], I will be allowed to disagree with his opinion, without losing even a minimum particle of that esteem, in which I deservedly hold the most erudite author. I said that the depth of the flat area, or valley, which may be observed at the foot of our hill, and that is commonly called the Garden [I Maggio Square], fits admirably with the tradition because as we were able to ascertain it is lacking much more earth than would now be required to form the whole height of the hill, in comparison with the remaining large plain where it is located.

On the grounds of this extraordinary lack of earth that forms the said valley, against the nature and perfection of the plain where it lies, and of the rotundity and uniqueness of the nearby hill, without there being any other eminence, even minimal, of earth in the vicinity, for the distance of many and many miles, who would not conclude that our hill was built by human skill and not by nature, and made with the very same soil which is clearly missing at its foot? Let it not be said that the interior of this hill is partly composed of solid rock, because this is not at all true, as has recently been seen in the excavation carried out in the very heart of the hill, on the occasion of rebuilding its ancient and deep water tanks, during which there was no sign whatsoever of stone or boulders in the sides of the excavations, but rather gravel and earth; This same thing has been observed previously, and can be seen throughout the hill from head to foot, apart from soft and fragile tufa [weakly cemented gravel] which commonly forms over time elsewhere near the surface of buildings and walls. This is indeed a beautiful monument, lasting and not subject to

being misinterpreted or altered, and cannot have been brought here from somewhere else, and is of such a great and distant antiquity, as to be distant from our gaze, and yet it is immersed and lies in the obscurity of past time; hence the awestruck common people over the course of time were unable to attribute this great enterprise to anyone else other than the most renowned Champions of antiquity; thereby clearly indicating to posterity the great antiquity of the place, which was then also recognized by the two famous restorers of ancient Geography, Cluverio and Cellario, and with them the above-mentioned illustrious Marquis Maffei."

Typo-chronology of the pottery assemblage from the "Fossa Bronzo" pit (1987 excavation)

The analysis of the conspicuous ceramic assemblage (about 8000 fragments) collected in 1987 from the pit excavated along the eastern edge of the Udine Castle Hill (Figs. S3 and S4B) was carried out by Giovanni Tasca. We refer to the recent publication where details about the typo-chronology of the finds are explained (*15*). Overall, the pottery assemblage is attributed to a period spanning from the beginning of RBA to the mature Early Iron Age (ca. 1350-600 BCE; see Fig. 5 and Supplementary information Fig. S5).

A significant group of pottery finds, including cups and bowls with rims of different shape, ovoid and bi-conical jars also decorated with plastic ornaments, are typo-chronologically dated to the RBA1 and RBA2 not advanced. Some few finds, including different types of handles, suggest a slightly older age based on comparisons with the pottery assemblage of the Karst and Istria castellieri, attributed to the transition from MBA to RBA [(# 8 and # 12 in Fig. 5 and Supplementary information # 10 and # 7 Fig. S5; (*15*)].

Later occupation on the hilltop is indicated by another substantial and better preserved group of vessels, including several bowls, jars and bi-conical vases dated to RBA2-FBA1 (1200-1100 BC), which show a broad spectrum of contacts with the communities of the Venetian Plain and the middle-Danubian groups (*15*). The handle decoration, with superimposed little tongue associated with bowls and pots with cylindrical neck (# 235 in Fig. 5), is considered a typical indicator of this phase (ca. 1200–1150 BCE) and represents a distinctive local input, presently known only in the contexts of the Friuli area [Fig. 5 and Supplementary information Fig. S5; (*41–42*, *11,15*)].

Less documented is the following phase of mature FBA (ca. 1100–1000 BCE), best signaled by some pottery fragments decorated with dots or dots and grooves that are largely spread in both Friuli and the Veneto coeval contexts [(# 343 in Fig. 5 and # 453 in Supplementary information Fig. S5; (*41–42*, *11,15*)].

Numerous cups and pots with cylindrical neck and bi-conical jars with everted and 'a tesa' rims can be referred to the FBA3-Early Iron Age (ca. 1000-800 BC) and show a clear link to the cultural groups of the middle and late phases of the Urnfield culture, particularly of the Slovenian area (*15, 43*). Finally, a small group of vessels, including some situla-shape and bi-conical jars with remarkable comparisons in the Venetian plain, testifies the occupation of the site within the mature Early Iron Age (around 800-600 BC; *15*).

Paleobotanic analysis

Palynostratigraphic data from a depth between 30.90 and 31.90 m in core CAST-2 show a general homogeneity of the microbotanical content, apart from the lowermost sample (31.85 m), where pollen and microcharcoal concentrations had their minimum values. Pollen of trees and shrubs (approx. 40–60%, Fig. S9) is mainly related to *Corylus*, *Alnus glutinosa* type and *Quercus robur-cerris* type. Upland herbs include all herbaceous taxa are unrelated to aquatic

environments (i.e., Apiaceae, *Aster* type, Cichorioideae and Ranunculaceae) and Poaceae. The latter is classified based on grain diameter [D] and *annulus* diameter [d] represent an important source of the pollen input, up to 40% at 30.95 m depth (Fig. S9). Poaceae with D>47 μm and d>11 μm are referred to cereals [*i.e.*, cultivated *Avena* and *Triticum* species, Cerealia type *sensu* (*36*)].

Synanthropic and cultivated taxa (red curves, Fig. S7) occur with relatively moderate values (<7%), but rather continuous curves. *Plantago lanceaolata* shows values around 1%, reaching approximately 5% in the sample at 30.95 m; *Orlaya grandiflora* type occurred in four samples (30.95, 31.25, 31.40 and 31.50 m), with mean values of approximately 0.7%. Cereals were detected almost continuously (approx. 0.3%), from 30.95 to 31.50 m.

Microcharcoal fragment concentrations (particles/cm^3) display mean values of 35,000 part./cm^3 (10–62 μm length class), with the highest value recorded in sample 31.70 m (approx. 54,000), even exceeding pollen concentration (approx. 25,000 grains/cm^3). By contrast, microcharcoal fragments and pollen concentrations dropped in the deepest sample (31.85 m depth) to approx. 2000 and 5000, respectively (Fig. S7).

Fern spores (trilete spores cfr. *Pteridium*) were the most abundant Non-Pollen Palynomorphs and were recorded with mean values of approximately 17%; Dung-related spores (*Sordaria* type and Sordariaceae undiff.) were present in low percentages in the record (Fig. S9). Such spores may be related to fungi developing in nutrient-enriched substrates possibly indicating the presence of grazing activities. The *Alnus glutinosa* type (alder) record, together with lowland and wetlands taxa (i.e., Cyperaceae, *Sparganium*, *Thalictrum*, *Potentilla* type), up to 2%, likely indicate the existence of a perilacustrine belt surrounding a water body close to the site. This is an extraordinary situation in the gravelly sector of the Friulian Plain, where the water table generally lies at a depth of 30–70 m. Thus, the peculiar hydrologic setting could justify the interest of ancient people at this site in Udine since remote periods.

Interestingly, the occurrence of *Orlaya grandiflora* in this sequence provides independent chronostratigraphic hints, since the record of this synanthropic taxon is well known in the area of the central and eastern Po Plain during the Bronze Age (*44*), although information is lacking for the Venetian and Friulian Plain. The fairly synchronous expansion of *Orlaya grandiflora* is related to the anthropogenic management of pristine forests, leading to open vegetation (i.e., cereal cultivation and pasture development) since 1870 ± 70 BCE at Lavagnone and 1800 ± 50 BCE at Lucone pile-dwelling villages (*44*).

Above the depth of 30.8 m in core CAST-2, microbotanical analyses were carried out on samples at 24.80–95, 18.65 and 12.15 m, taken from weak-organic intervals between gravel and clayey lenses alternations. The poorly preserved palynomorph assemblages from these samples suggest a microbial and/or chemical degradation of organic remains, apart from millimetric charcoal. Such material was radiocarbon dated obtaining three ages in chronologically reversed order between 4330 and 1770 years BCE, suggesting that the sediments had been reworked from older deposits.

Stratigraphy, morphology and chronology of the mound

Based on the available set of radiocarbon dates and archaeological evidence, the Udine castle hill mound sealed a previous surface that was exposed at least until about 1300 BCE and its construction buried a soil that had developed over an almost flat surface with strong evidence of previous human occupation. The most significant information about the ancient existence of the mound and its height of 30 m is the pit found in 1987 during excavation of the eastern edge of

the hilltop and named "Fossa Bronzo" [Bronze-Age pit; (*14*)]. The earliest pottery fragments found in it document its existence and the occupation of the hilltop from the end of RBA. It remains difficult to propose a reliable hypothesis on the construction time of the mound, as it may have varied from a few decades to more than a century, depending on the number of people involved in the construction and the frequency and length of the working periods over the course of the year.

A major and significant characteristic of the prehistoric mound is the large top area that already existed during its initial phase. On the basis of all the data collected in the eastern sector of the hilltop (1986–87 and 2021 archaeological excavations, core CAST-5 near the Castle mansion and the excavations for the water tanks dug in the 1950s), it is evident that prehistoric layers were found at a shallow depth most of the esplanade. Although minor repair works could have been carried out on the mound during the Iron Age, when the fortified site at Udine continues to constitute the main center of the Friulian Plain, they most probably involved only limited sectors of the structures. At those points where the construction of the modern water tanks has not destroyed the top portion of the archaeological stratigraphy, traces of medieval-Roman occupation were found over the entire hilltop, directly reworking and/or covering the prehistoric structures or the landfill deposits forming the mound. Despite the poor conservation of the prehistoric surface on the top of the mound, the archaeological data gathered elsewhere within the *castelliere* of Udine documents that the area was also settled during the Iron Age and that the fortified settlement lasted until the very arrival of the Romans, suggesting continuity of settlement.

Radiocarbon dating carried out on the charcoal samples collected in the horizons with weak organic content found within the body of the mound documented that some of the fine sediments used for the erection of the mound came from the top portion of the soil existing in the surrounding alluvial plain. In particular, the dates were clearly in reverse chronological order. Moreover, in the 2021 archaeological excavation, a charcoal found within one of the gravelly lenses (US 107) was almost 3000 years older than the base of the mound itself. The occurrence of many charcoals predating the mound is compatible with anthropogenic activities caused by forest clearance, fires and territorial management carried out in the area since the Neolithic.

Based on the available data and the absence of similar contemporary structures, it remains difficult to speculate about the function of the mound. The convex morphology of the Udine mound leads us to compare it with the burial tumuli that have been widely documented in the Friulian Plain (Figs. 1c and 6), built since the EBA. Notwithstanding, among these latter monumental earthen constructions, the largest reach a volume between 5000 and 10,000 m^3 (*45*), which is a degree of magnitude smaller than the anthropogenic mound of Udine. This suggests that the mound of Udine had a different function than a burial monument. The artificial hill surely became a very important landmark in a completely flat area. According to its dates, the construction of the mound of Udine coincided with a phase of great changes in northern Italy, as well as at a Mediterranean scale. In the Friuli area, this included a profound reorganization of the settlement system documented by important rebuilding work on the fortifications at several *castellieri* and the abandonment of many previously occupied sites in the further plain (Fig. 1c). Therefore, it is reasonable that the construction was connected to these events.

The technique used for the landfill that constitutes the Udine Castle Hill was described in detail during the 2021 excavation in the eastern hilltop. It consists of alternating layers of loamy gravels and rubified clays with the occurrence of wooden buttresses. These alternating gravelly and clayey lenses were clearly documented by both the stratigraphic cores and the stratigraphic

evidence from the wartime tunnels dug in 1943 at the base of the mound. The same technique was used for the entire height of the mound, of about 30 m. Interestingly enough, this corresponds to the technique used when the ramparts of the Udine fortified settlement were rebuilt, as documented at the foot of Udine castle hill at Palazzo Mantica (*46*) and, for the same purposes, at other *castellieri* in the Friuli Plain, including Sedegliano, Galleriano and Savalons (see Figs. 1c and 6 for the location and morphology of these sites). On the basis of the pottery assemblages associated with this rebuilding phase, the construction works are likely to have occurred simultaneously in those sites at the end of RBA (*11, 47, 41*). This technique is functional in reducing landslide processes resulting from the soaking of fine sediments, and is documented by ethnographical studies and experimental projects (*48–50*).

The use of similar techniques is also known in other cultural contexts. Gabion structures are known in the terramare at Castione of Marchesi and Gaggio (*51–53*). Settlement fortifications consisting of wooden boxes filled with rubble are attested in central-eastern Europe at several settlements of the Lausitz culture (*54*). It is possible that the development of new construction techniques is a consequence of the unprecedented growth of trade and exchange networks in the later Bronze Age and the overall interconnectedness of communities at the European scale (*55–56*).

Estimation of the mound volume

The stratigraphic data demonstrate that the elevation of the natural ground surface pre-existing the artificial Udine Castle Hill ranges between approximately 110 and 111 m asl, with some topographic peaks up to 112 m asl. Based on this information, we estimated the volume of sediment forming the current mound according to three different scenarios (see Fig. S9). Based on our calculations, the Udine mound currently has a volume between 600,000 and 650,000 m^3 and between 350,000 and 450,000 m^3 of sediment was extracted from I Maggio Square. During historical periods, the mound underwent some minor works that enlarged the top portion of the hill and partly expanded its base. A major result of this work was the additional perimeter containing wall that encircles the hilltop and extends the radius by 5–10 m of the esplanade.

A minimum estimate of the volume of the prehistoric mound can be made by considering a cone shape with a basal radius of 100 m and a height of 30 m. This would result in an overall volume of 314,000 m^3 (Fig. S9). However, considering the topographic, stratigraphic and chronological data, this hypothesis is highly unlikely and strongly underestimates the real volume. According to the available stratigraphic data, it is likely that the top of the hill had a flat surface since it was built and had a circular shape with a radius of between 30 and 50 m. A radius of 35 m can be considered a conservative estimate.

Any hypothesis that the prehistoric mound was higher than 30 m from its base would imply that it had a larger volume. Although this hypothesis cannot be ruled out, the archaeological and stratigraphic data collected in the eastern sector of the hilltop (Fig. 5) suggest that, at least in some portions, an overall hilltop elevation of around 141 m asl was likely, so it was about 30 m high.

Another geometric factor affecting the estimated volume is the geometry of the slopes of the mound, which could have been either inclined or stepped (Fig. S9c). If the slopes were inclined, the geometric solid that simplifies the mound would be a truncated cone. In this case, a volume calculation based on a 35 m radius for the hilltop and 100 m for its base would result in about 462,000 m^3 (Fig. S9b).

The highest earthen constructions dating to RBA and FBA in NE Italy [e.g., the tumulus of Mereto (*57*)] have stepped slopes; it is therefore likely that the slope of the mound had a stepped form. Following this hypothesis, one of the simplest simulations divides the total height of 30 m into 5 steps of 6 m each, which would mean that the mound volume would have been 386,000 m^3 (Fig. S9c). However, this hypothesis of an earthen construction with vertical scarps of 6 m would have been geotechnically weak; it is more likely that the vertical steps were subdivided in shorter intervals, of about 1.5 m. This hypothesis is illustrated in Fig. S9d, and implies a volume of approximately 404,000 m^3.

Analyzing all possible scenarios, the volume of the prehistoric mound was probably between 378,000 to 462,000 m^3. We stress that these estimates err on the side of caution, and higher values of up to 550,000 m^3 are possible. In all these simulations, the Udine mound has a volume that was larger than that calculated for Silbury Hill, which, up to now, was believed to be the largest artificial prehistoric mound in Europe. The Silbury Hill mound measures about 40 m in height, 80 m in radius at its base and has an overall volume of nearly 350,000 m^3 of earth and chalk blocks that have been quarried from the area at the base of the hill (*58*).

Total Viewshed Analysis

The results of the Total Viewshed Analysis indicate that: (i) the mounds and the *castellieri* are more visible and have greater visibility than all the other random sites located in their surroundings; (ii) that the top of the Udine Castle Hill is far more visible and also has a larger viewshed than other locations in the central portion of the Friulian Plain.

In more detail, the mean visibility index at different radius (10, 20 and 30 km) indicates that the *castellieri* of Pozzuolo and Variano (see the chart in Fig. S10) are slightly more visible than the other considered sites. This is not surprising considering that these fortified settlements were built on the top of natural tectonic reliefs to guarantee a better protection from possible raids and to enhance, at the same time, their visibility. However, it is possible that these two settlements were only partly visible from a distance given their relatively low elevation from the surrounding plain (i.e., 10–15 m) and the likely presence of woodland. In contrast, it appears to be significant that the visibility from the Udine Castle Hill is greater than that from all the other considered sites.

The results obtained from the Monte Carlo simulation strongly indicate that mounds and fortified settlements are more visible and at the same time have greater visibility than all the other random points located within the convex hull (p-value 0.01). In the second step, we compared the visibility index at a 30 km radius of 100 random points collected on the top of the Castle Hill of Udine with 99 samples of 100 random points located within the entire investigated area. Interestingly enough, results strongly indicate that the top of the hill of Udine is more visible and at the same time has a larger visibility than the background population (p-value 0.01; Fig. S10).

Toponyms and folklore traditions related to Attila the Hun and other hills in NE Italy

The name of Attila is documented in legends and toponyms in several locations in Northern Italy, especially in the regions of Friuli and Veneto. His name is sometimes attached to natural features with bizarre shapes (e.g., cave entrances that look like a skull or mountain crests with a chair shape). In other cases, the name of Attila the Hun is connected to popular histories that are not closely related to landforms, for example, those that tell of treasures hidden by the Huns or alternatively by the local people to avoid their theft during the invasion. Thus, we concentrated

our research on the places in NE Italy where the name of Attila is attached to hills and mounds, and this approach led us to consider Col d'Attila, near Farra di Soligo, the Bastione of Attila, near Lugugnana of Portogruaro, and Monte Purga in the Lessini Mountains north of Verona (Fig. 1c).

There is a small mound near the town of Lugugnana of Portogruaro, along the northern fringe of the area that was formerly part of the Caorle Lagoon, reclaimed at the beginning of the 20th century. This mound is also called the "Mottaron", meaning a large "motta", a local name for a mound. In this area, the tradition was that this mound was Attila's bastion, as also reported by the famous writer Ippolito Nievo in chapter three of his novel "Le confessioni di un italiano", printed in 1867. The original text is: "*Io poteva essere stato al Bastione di Attila, che è un'altura presso la marina di fianco a Lugugnana dove la tradizione paesana vuole che venendo da Aquileia abbia tenuto suo campo il re degli Unni prima di essere incontrato dal pontefice Leone.".* Nievo reports the local tradition that the mound was built by the Huns as a camp on the way to meet Pope Leone.

The small hill is clearly shown on the topographic map surveyed in 1798–1805 under the direction of General Anton von Zach, that is generally called the Kriegskarte (*59*). The artificial structure was leveled soon after First World War, when the main phase of reclamation took place. As can be seen on the map shown in Fig. S12, the mound is isolated, and reached a height of about 5 m. Currently, there are no clues concerning the date of construction of the of Lugugnana mound, but its artificial origin is clear.

There is a significant toponym near Farra di Soligo, at the mouth of the Piave River valley, where two isolated hills are named Colle d'Attila (Hill of Attila) and Col della Regina (hill of the Queen). Colle d'Attila is the highest of the two, rising about 20 m above the surrounding plain. The origin of the reliefs is still debated and the possible explanations are do not fit well with the geomorphological context of the area. In this case it is not clear that they are artificial and a small convex natural landform was probably present. According to the popular tradition, the mound was a tumulus built by the Huns for their king, whose soldiers used sediment dug from the bed of the river Piave to divert it and drain a former lake, demonstrating that nothing was impossible for Attila.

Further away, in the Lessini Mountains, in the Pre-Alps north of Verona, a folktale describes the top of Mount Purga (Fig. 1D) as a place where Attila and his hordes built a temporary fort during their retreat from Italy because of the advantage given by its elevated position dominating the valleys. Remains of an Iron-Age hillfort and historical structures have been recorded at this site (*60*).

Data S1. (separate file)
Stratigraphic description and log of core CAST-2.

References

1. Frigingensis, O. *Ottonis episcopi Frisingensis Chronica : sive, Historia de duabus civitatibus*, ed. Adolf Hofmeister (Hahnsche Buchhandlung, 1912)
2. Muratori, L. *Rerum Italicarum Scriptores Ab Anno Æræ Christanæ Quingentesimo Ad Millesimumquingentesimum Quorum Potissima Pars Nunc Primum In Lucem Prodit Ex Ambrosianæ, Estensis, Aliarumque Insignium Bibliothecarum Codicibus T. 7* (Mediolani ex typographia Societatis Palatinae, 1725).
3. Marin Sanudo il Giovane, *Descrizione della patria del Friuli*. (Premiata Tipografia di Pietro Naratovich, 1853).
4. da Valvasone, E. *La caccia dell'ill sig. Erasmo di Valvasone, ricorretta & dimolte stanze ampliata.* (per Fran. Bolzetta, 1602).
5. Albrizzi, G. *La Patria Del Friuli; Descritta Ed Illustrata Colla Storia E Monumenti Di Udine Sua Capitale E delle altre Città e Luoghi Della Provincia*. (Stamperia Albrizzi, 1753).
6. Fistualario, P. & Valvasone, J. *Monografie Friulane offerte a Monsignore Zaccaria Bricito, arcivescovo di Udine ece.* (Vendrame, 1847)
7. Visentini, P., Borgna, E., Borzacconi, A., Buora, M., Cividini, T., Corazza, S., Musina, G., Petrucci, G., Pizziolo & Tasca, G. Il progetto “Archeologia urbana a Udine”: le prime indagini in via Mercatovecchio (1989). *Gortania* **43**, 75–142.
8. FAO-ISRIC, *World reference base for soil resources 2006: a framework for international classification, correlation and communication*. (FAO, 2006).
9. Munsell Color, *Munsell Soil Color Chart* (Munsell, Baltimore, USA, 1994).
10. Cardarelli, A. The Collapse of the Terramare Culture and growth of new economic and social Systems during the Late Bronze Age in Italy. *Scienze dell’Antichità* **15**, 449-520 (2009).
11. Borgna, E., Càssola Guida, P., Corazza, S., Mihovilić, K., Tasca, G., Teržan, B. & Vitri, S. Il Caput Adriae tra Bronzo Finale e antica età del Ferro in *Preistoria e Protostoria del Caput Adriae* (eds. Borgna, E., Càssola Guida P., & Corazza, S.) 97–118 (Istituto Italiano di Preistoria e Protostoria, 2018b).
12. Hänsel, B. Die Bronzezeit 2200-800 v. Chr. in *Atlas der Vorgeschichte. Europa von den ersten Menschen bis Christi Geburt*, (ed. Von Schnurbein, S.) 108-149 (Theiss, 2009).
13. de Marinis, R. C. Towards a relative and absolute chronology of the Bronze Age in Northern Italy. *Notizie Archeologiche Bergomensi* **7**, 23–100 (1999).
14. Buora, M. Udine - Scavi sul colle del castello. *Aquileia Nostra* **58**, 335-342 (1987).
15. Tasca, G., La fossa protostorica. Archeologia Urbana a Udine 2: Una rilettura dei dati provenienti dal colle del Castello. *Monografie Museo Friulano Storia Naturale* **48**, 55-166 (2023).
16. Bernardis, G. & Zorzi, P. *Studio geologico-tecnico in prospettiva sismica del territorio comunale.* (Comune di Udine, 1981).
17. Bernardis, G. & Zorzi, P. Le problematiche geologiche del colle del castello di Udine. *Rassegna Tecnica Friuli Venezia Giulia 38(6)*, 28–30 (1989).
18. Comel, A. I terreni dell’anfiteatro morenico del Tagliamento e dell’alta-media pianura del Friuli centro-orientale. *Annali Staz. Chim. Agr. Sperim*. Udine, s. 3, **6**, 1–89 (1939).
19. Comel, A. Probabile origine morenica del lembo prewürmiano situato nei pressi della stazione ferroviaria di Udine. *Nuovi Studi Staz. Chim. Agr. Sperim. Udine* **37**, 1–7 (1961).

20. Comel, A. Presenza di erratici nel centro di Udine. *Boll. Soc. Adriatica di Scienze* **52** (II), 1–7, Trieste (1962).
21. Comel, A. Il masso erratico di Via Poscolle in Udine. *Nuovi Studi Staz. Chim. Agr. Sperim. Udine* **65**, 1–5 (1964).
22. Comel, A. Configurazione morenica del sottosuolo di Piazzale Osoppo in Udine. *Nuovi Studi Staz. Chim. Agr. Sperim. Udine* **74**, 1–3 (1965).
23. Comel, A. Due importanti documenti sulla transizione della morena prewürmiana ferrettizzata di Udine verso le contigue alluvioni würmiane. *Nuovi Studi Staz. Chim. Agr. Sperim. Udine* **90**, 1–6 (1969).
24. Feruglio, E. A proposito della costituzione geologica del colle di Udine. *In Alto* **31**, 18–21 (1921).
25. Feruglio, E. *Note illustrative della Carta geologica delle Tre Venezie: Foglio Udine* (Uff. Idrogr. R. Magistrato Acque, 1929).
26. Lorenzi, A. Sulla natura geologica del colle di Udine. *In Alto* **31**, 16–18, (1920).
27. Martinis, B. Geologia del colle e del sottosuolo di Udine. *Ist. Geol. Paleont. Geogr. Fis., ser. G* **74**, 1–49, (1953).
28. Paronuzzi, P. Stratigrafia e sedimentologia di una sequenza fluvioglaciale würmiana del centro di Udine. *Il Quaternario – Italian Journal Quaternary Sciences* **1**(2), 111–126 (1988).
29. Zorzi, P. Lineamenti geomorfologici, in *Piazza Primo Maggio a Udine. Spazio e Architettura, Storia di uno spazio urbano in cerca di identità* (ed. Biasi A.) 13-32 (Forum, 2008).
30. Zanferrari, R., Avigliano, G., Monegato, G., Paiero, M. & Poli, E. (eds.) *Note illustrative della Carta Geologica d'Italia alla scala 1:50.000 – Foglio 066 "Udine"* (Regione Friuli Venezia Giulia, 2008).
31. Stuiver, M., Reimer, P. J. & Reimer, R. W. *CALIB 8.2.* http://calib.org (2021).
32. Reimer, P.J., et alii, The IntCal20 Northern Hemisphere radiocarbon age calibration curve (0–55 cal kBP). *Radiocarbon* **62***(4)*, 725–757 (2020).
33. Stockmarr, J. *Tablets with spores used in absolute pollen analysis* (1970). Available at: https://www.scienceopen.com/document?vid=d49fdd1d-5f59-48b6-8c6d-99fef455836e (Accessed: 31 March 2020)
34. Beug, H. J. *Leitfaden der Pollenbestimmung fur Mitteleuropa und angrenzende Gebiete.* (Verlag Dr. Friedrich Pfeil, 2004).
35. Punt, W., Blackmore, S., Clarke, G.C.S., Hoen, P.P. & Stafford, P. J. *The Northwest European Pollen Flora. voll. I-IX.* (Elsevier, 1976).
36. Reille, M. *Pollen et Spores d'Europe et d'Afrique du Nord. Laboratoire de Botanique Historique et Palynologie* (Laboratoire de botanique historique et palynologie, 1992).
37. Reille, M. *Pollen et spores d'Europe et d'Afrique du Nord–Supplément II* (Laboratoire de botanique historique et palynologie, 1998).
38. Joly, C., Barillé, L., Barreau, M., Mancheron, A. & Visset, L. Grain and annulus diameter as criteria for distinguishing pollen grains of cereals fromwild grasses. *Rev. Palaeobot. Palynol.* **146**, 221–233 (2017).
39. Clark, J.S. Particle motion and the theory of charcoal analysis: Source area, transport, deposition, and sampling, *Quaternary Research* **30**(1), 67–80 (1988).
40. Grimm, E. Tilia and TGView 19 version 2.0. 41. software. Springfield, USA: Illinois State Museum, Research and Collection Center (2015).

41. Tasca, G., Putzolu, C. & Vicenzutto, D. Between Po plain and middle Danube Urnfield cultures: Codroipo and the Friulian plain in XIIth century B.C in *Inter-regional contacts during the first millenium B.C. in Europe: proceedings from the session organized during the 19th meeting of European Association of Archaeologists, held in Pilsen (5th-9th September 2013)*, (eds. Trefný, M. & Trefný, B.) 4-23 (Hradec Králové, 2017).
42. Corazza, S. Nuovi dati sul Bronzo finale iniziale dalla pedemontana pordenonese, in *Protostoria e Storia del 'Venetorum angulus'* (ed. Paoletti, O.) 117-130 (Istituti editoriali e poligrafici internazionali, 1999).
43. Cardarelli, A. Castellieri del Carso e dell'Istria: cronologia degli insediamenti tra media età del bronzo e prima età del ferro, in *Preistoria del 'Caput Adriae'*, (eds. Boiardi, A. & Bartolomei, G.) 87-112 (Ist. per l'Enciclopedia del Friuli Venezia Giulia, 1983).
44. Perego, R., Badino, F., Deaddis, M., Ravazzi, C., Vallè, F. & Zanon, M. L 'origine del paesaggio agro-pastorale in nord Italia: espansione di Orlaya grandiflora (L.) Hoffm. nella civiltà palafitticola dell'età del Bronzo della regione del Garda. *Notizie Archeologiche Bergomensi* **19**, 161–173 (2011).
45. Vinci, G. & Calosi, M. Tumuli e castellieri. Tecniche di analisi spaziale applicate allo studio del paesaggio monumentale della pianura udinese del II millennio a.C. in *Dall'Adriatico all'Egeo. Scritti di Protostoria in onore di Paola Càssola Guida* (eds. Borgna, E. & Corazza, S.) 61-70 (Forum, 2021).
46. Vitri, S., Borzacconi, A., Corazza, S., Simeoni, G., Marchesini, M. & Petrucci, G. Palazzo Mantica. Resti protostorici e bassomedievali/rinascimentali. *Notiziario della Soprintendenza per i Beni Archeologici del Friuli Venezia Giulia*, 4/2009, 46-57 (2013).
47. Corazza, S. I sistemi difensivi dei castellieri del Friuli: cronologia e modalità costruttive in *Preistoria e Protostoria del Caput Adriae* (eds. Borgna, E., Càssola Guida P. & Corazza S.) 209-222 (Istituto Italiano di Preistoria e Protostoria, 2018).
48. Vicenzutto, D. *Il fenomeno degli abitati fortificati di pianura dell'età del bronzo nell'Italia settentrionale a nord del Po: terramare, siti arginati e castellieri* (University of Padua, Italy, 2017).
49. Bordoni, A. *Degli argini di terra* (Paolo Emilio Giusti, 1820).
50. Bell, M., Fowler, P. J. & Hillson S.W (eds). *The experimental earthwork project, 1960-1992.* (Council for British Archaeology, 1996)
51. Cupitò, M. Dinamiche costruttive e di degrado del sistema aggere-fossato della terramara di Castione dei Marchesi (Parma). Rilettura e reinterpretazione dei dati ottocenteschi. *Rivista di Scienze Preistoriche* **62**, 231–248 (2012).
52. Cardarelli, A. Centri fortificati dell'età del bronzo in Italia centro-settentrionale in *Mura di legno, mura di terra, mura di pietra: fortificazioni nel Mediterraneo antico* (eds. Bartoloni, G. & Michetti, L. M.) 19–43 (Quasar, 2013).
53. Cardarelli, A. Before the city: the last villages and proto-urban centres between the Po and the Tiber rivers. *Origini* **42**(2), 359-382 (2018).
54. Coblenz, W. Die Burgwalleun das ausklingen der westlichen Lausitzer Kultur in *Symposium zu Problemen der jüngeren Hallstattzeit in Mitteleuropa*, (ed. Chropovsky, B.) 85-99 (Veda, 1974).
55. Iacono, F., Borgna, E., Cattani, M., Cavazzuti, C., Dawson, H., Galanakis, Y., Gori, M., Iaia, C., Ialongo, N., Lachenal, T., Lorrio, A., Micó, R., Molloy, B., Nafplioti, A., Peche-Quilichini, K., Rihuete Herrada, C. & Risch, R. Establishing the Middle Sea: The Late Bronze Age of Mediterranean Europe (1700–900 BC). *J. Archaeol. Res.* (2021).

56. Kristiansen, K. The Rise of Bronze Age Peripheries and the Expansion of International Trade 1950–1100 BC in *Trade and Civilisation: Economic Networks and Cultural Ties, from Prehistory to the Early Modern Er*a, (eds. Kristiansen, K., Lindkvist, T. & Myrdal, J.) 87-112 (Cambridge University Press, 2018).
57. Borgna, E., Simeoni, G. & Vinci, Origin and Evolution of a Bronze Age Funerary Landscape in Friuli: The "Lower Context" of the Tumulus of Mereto di Tomba (Udine) and the 3rd-2nd Millennium Transition in the Northern Adriatic. *Origini* **53**, 113–140 (2019).
58. Field, D., Leary, J. & Marshall, P. Neolithic Silbury in context in *Silbury Hill: The largest prehistoric mound in Europe* (eds. Leary, J., Field, D. & Campbell, G.) 223-254 (English Heritage, 2013).
59. Rossi, M. *Kriegskarte 1798-1805: il Ducato di Venezia nella carta di Anton von Zach = das Herzogtum Venedig auf der Karte Antons von Zach. 2 Descrizioni militari*. (Fondazione Benetton, 2005).
60. Zorzi, F. Il castelliere di M. Purga di Velo Veronese, *Atti dell'Accademia di Verona*, serie V, ***26***, (1950).
61. Nievo, I. *Le confessioni di un ottuagenario*. (Le Monnier, 1867).
62. Pacciarelli, M. *Dal villaggio alla città. La svolta protourbana del 1000 a.C. nell'Italia tirrenica.* (All'Insegna del Giglio, 2001).
63. Borgna, E., Càssola Guida, P., Mihovilić, K., Tasca G. & Teržan, B. Il Caput Adriae tra Bronzo Antico e Bronzo Recente in *Preistoria e Protostoria del Caput Adriae* (eds. Borgna, E., Càssola Guida P., & Corazza, S.) 75–96 (Istituto Italiano di Preistoria e Protostoria, 2018).

Figures and Tables

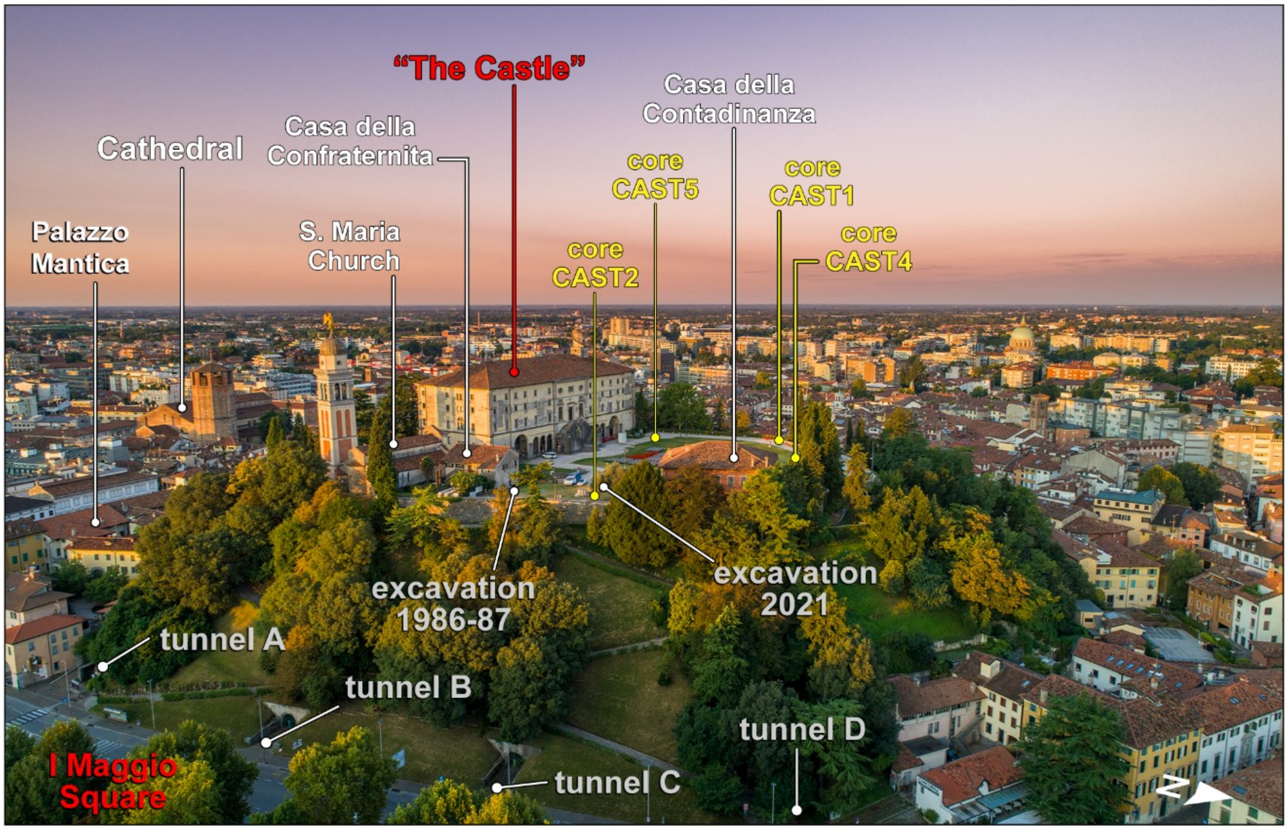

Figure S1. Oblique aerial photo of the hill of Udine from north-east to south-west (photograph by Fabio Pappalettera, DRONE REPORTAGE). The sites of the new stratigraphic cores drilled for this research from the top of the hill are indicated in yellow. The white writings indicate archaeological excavations and historical places in the city center of Udine.

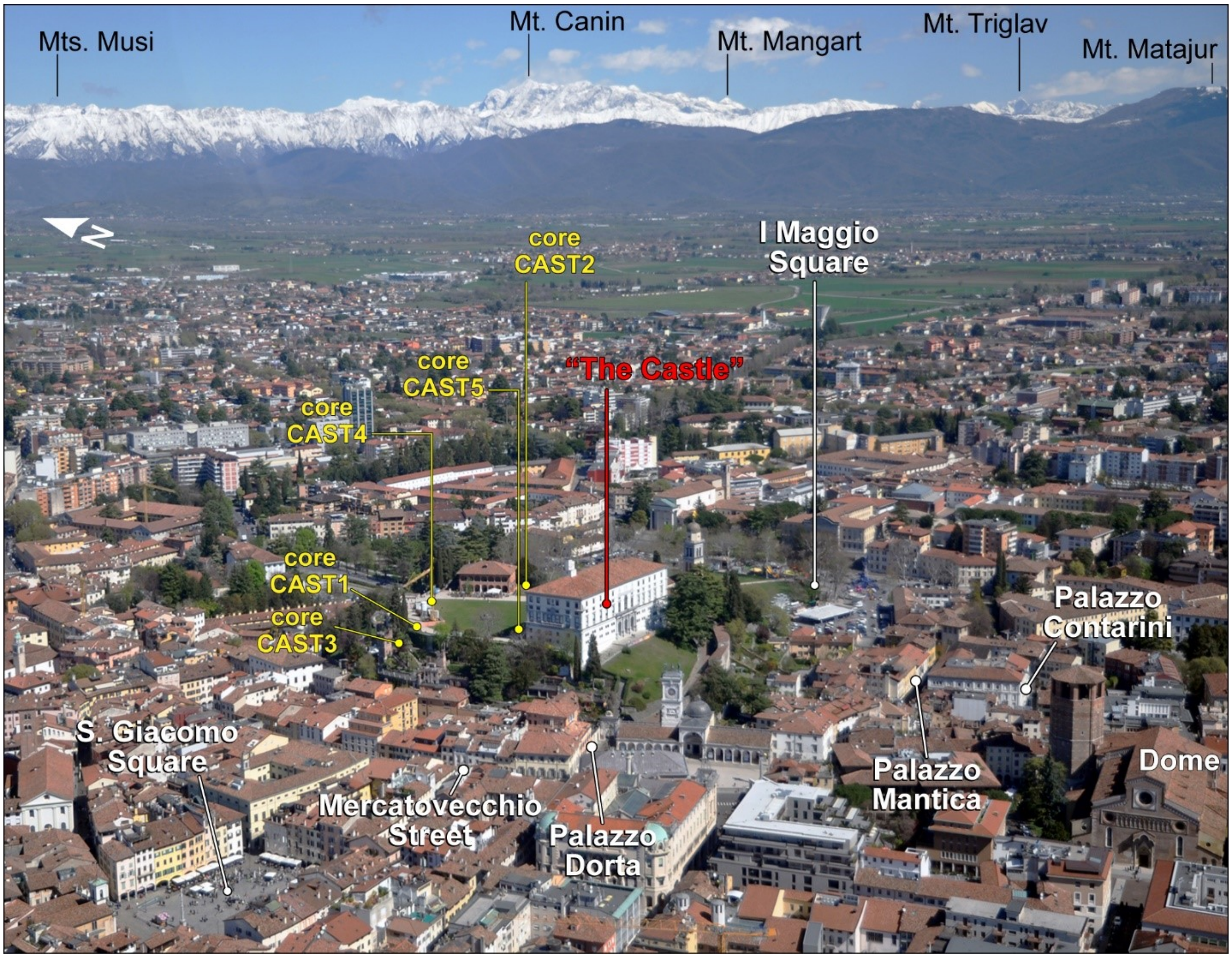

Figure S2. Oblique aerial photo of the hill of Udine from SW to NE (picture by Alessandro Fontana). The sites of the new stratigraphic cores drilled for this research from the top of the hill are indicated in yellow. The white writings indicate archaeological excavations and historical places in the city center of Udine.

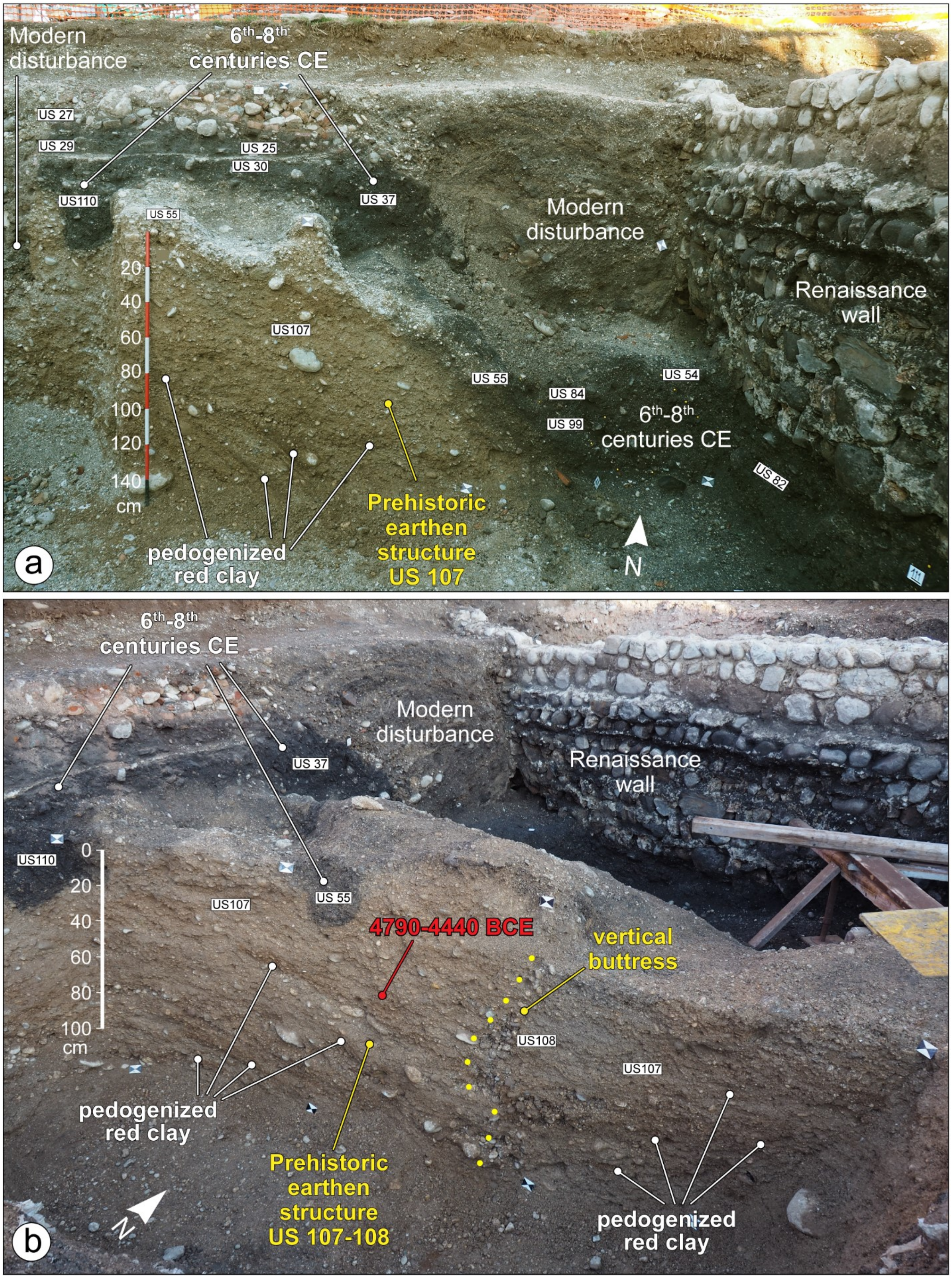

Modern disturbance
6th-8th centuries CE
US 27
US 29
US 25
US 30
US110
US 37
US 55
Modern disturbance
Renaissance wall
US107
US 55
US 54
US 84
US 99
6th-8th centuries CE
US 82
20
40
60
80
100
120
140
cm
pedogenized red clay
Prehistoric earthen structure US 107
N
a
6th-8th centuries CE
Modern disturbance
US 37
Renaissance wall
US110
0
20
40
60
80
100
cm
US107
US 55
4790-4440 BCE
vertical buttress
US108
US107
pedogenized red clay
Prehistoric earthen structure US 107-108
N
pedogenized red clay
b

Figure S3. Reference stratigraphic sections of the archaeological excavation carried out between October and December 2021, near the building named "Casa della Contadinanza" on the top of the hill of Udine (pictures by Alessandro Fontana). The radiocarbon date of a charcoal is reported in red; the old age reported demonstrates the use of reworked sediments.
(a) Section along the northern boundary of the investigated area, where the prehistoric earthen structure is clearly recognizable by the yellowish color of the alternations of gravels with reddish clays. The black deposits correspond to organic-rich silt with gravels and archaeological fragments dating between 5th to 8th century BC. In particular, US30 and US25 are ground floors, whereas US110 and US37 are pits eroding the prehistoric structure. The US55 has an erosive boundary and, together with US 84, 99 and 54, they are resting over the slope that is dipping eastwards. Over US54, two medieval tombs were found that were dated to 5th to 8th century BC. The wall on the eastern side had a Renaissance age and partly cut the medieval units. The modern disturbance near the wall was produced by the excavation of a trench of a water pipeline.
(b) Picture of a section from NW to SE across the prehistoric earthen structure. The drawing of this section is depicted in Fig. 2b. The alternation of gravel and clay lenses is clearly visible and marked also by the iso-orientation of major pebbles. Near the middle part of the section, a fairly vertical feature is present and characterized by clasts and pebbles with an open-work structure without matrix. This element is comparable to the wooden buttress recognized in the rampart bounding the settlement of Udine during the Bronze Age, as documented at the site of Palazzo Mantica (*46*) and other castellieri and tumuli located in the Friulian Plain (*47*).

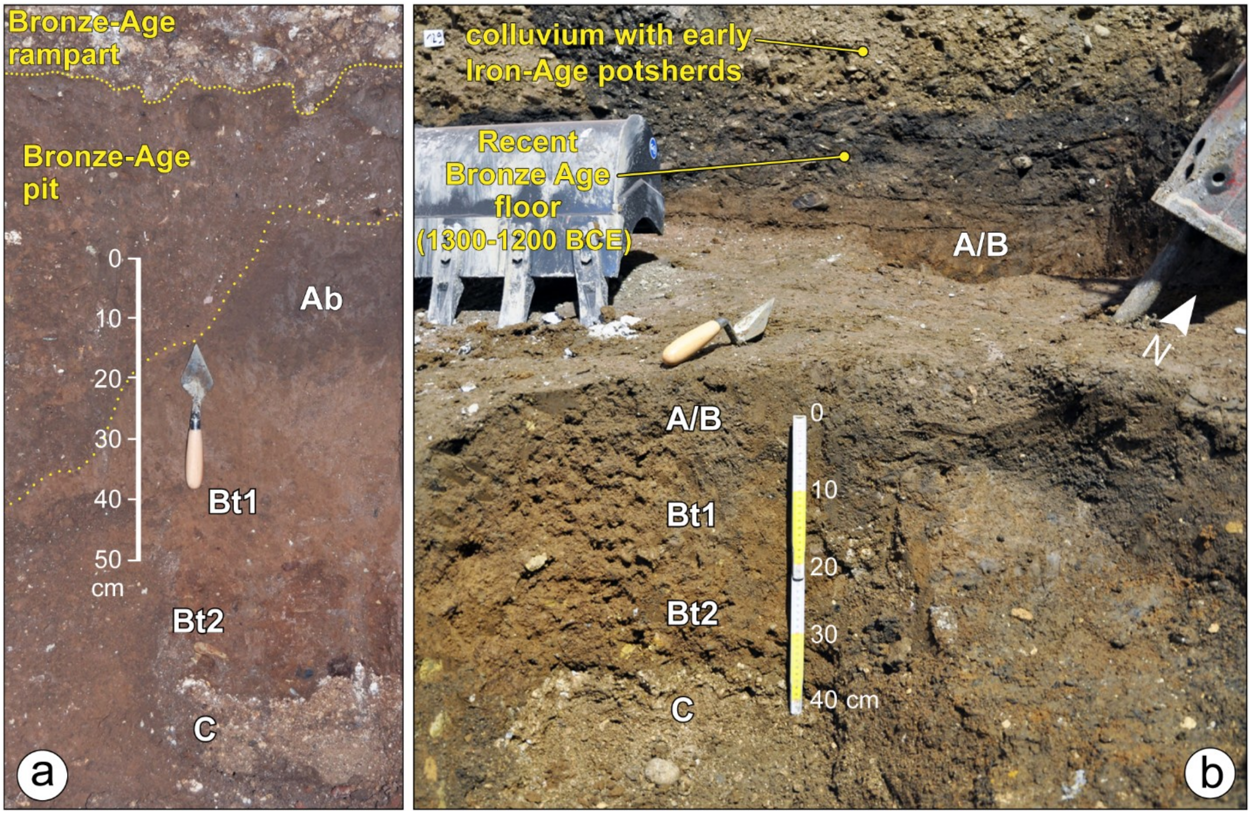

Figure S4. Pedologic profiles of the natural surface existing in the surroundings of the hill of Udine. See Fig. 2 for the location (pictures by Alessandro Fontana).
(a) Archaeological excavation of Palazzo Contarini, near Piazzetta Valentinis, in April 2022. The area was sealed by the construction during the Bronze Age of the rampart surrounding the settlement of Udine. The Bt2 horizon is very evident by its color and firm consistency, whereas Bt1 is looser and lighter. (b) Archaeological excavation of Palazzo Dorta, near Libertà Square, at the western toe of the castle hill. The drawing of this section is depicted in Figure 5c; see Figure 2 for the location. The natural surface is currently located at about 4 m of depth underground on the present street ground and had been sealed by a ground floor of a hut built during the Recent Bronze Age (ca. 1300–1200 BCE). The colluvium layers covering the Bronze-Age layer at the base includes some centimetric fragments of pottery dating to the early Iron Age. The colluvium dipped from east to west, representing an external expansion of the mound of the castle.

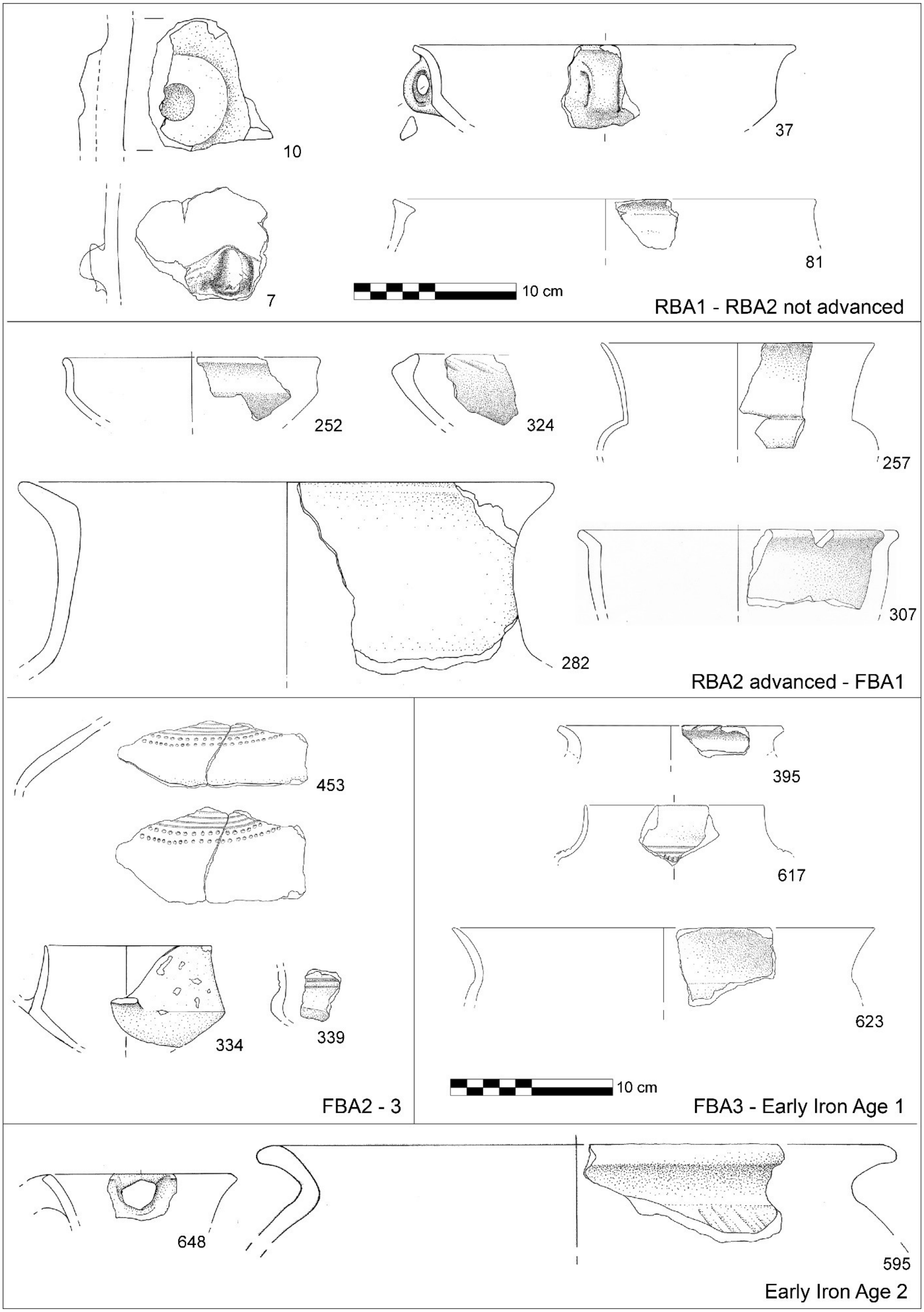
10
37
7
81
10 cm
RBA1 - RBA2 not advanced
252
324
257
307
282
RBA2 advanced - FBA1
453
395
617
623
334
339
10 cm
FBA2 - 3
FBA3 - Early Iron Age 1
648
595
Early Iron Age 2

Figure S5. Selection of some pottery fragments found in the "Fossa Bronzo" (Bronze-Age pit), excavated in 1987 in the eastern sector of the hilltop, near the building named “Casa della Confraternita”. See Figure 4 and S5b for the location. The description of the chrono-typological phases of the fragments is reported in the main text and in the supplementary text according to Tasca (*15*).

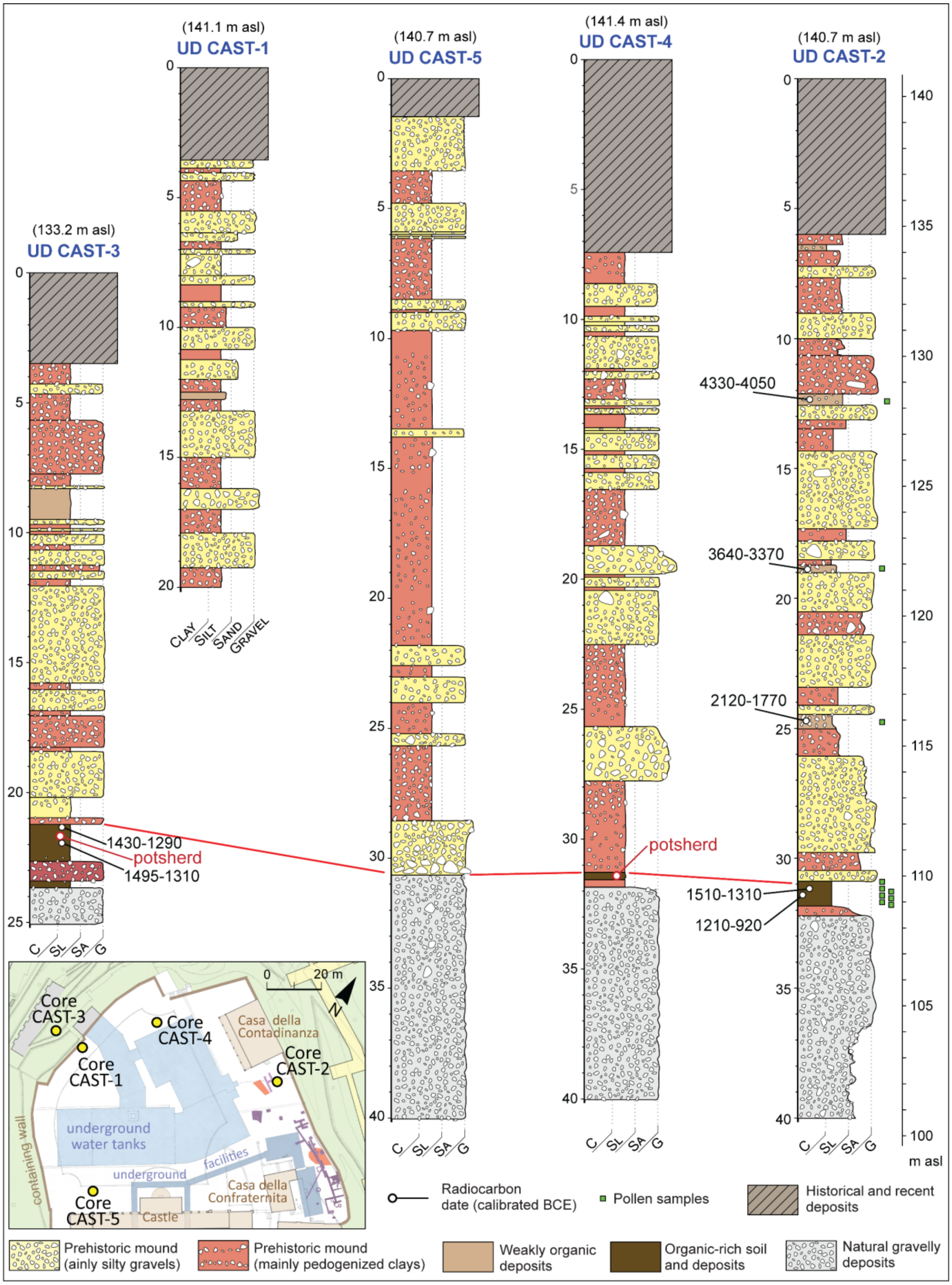

Figure S6. Stratigraphic logs of the cores carried out from the top of the hill of Udine between 2020 and 2022. It is worth noting the almost flat topography of the surface on which the mound was built, probably exposed until around 1400 BCE.

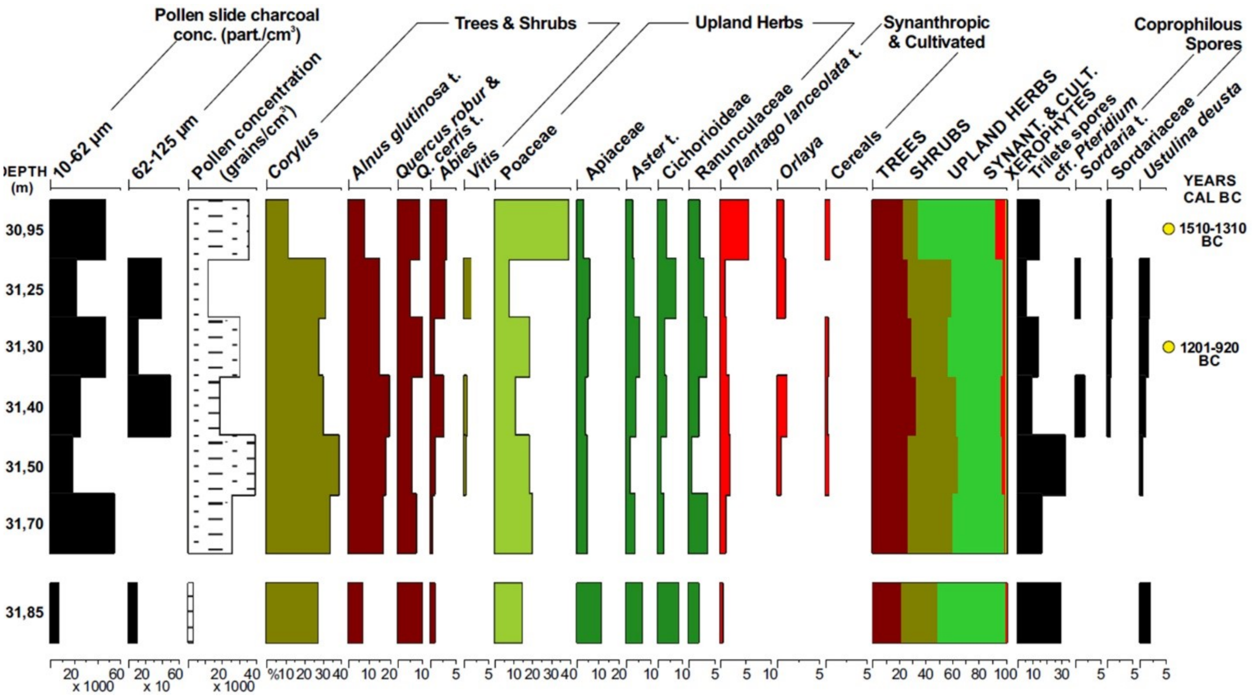

Figure S7. Plot diagram of the pollen data from samples between 30.8 m and 31.9 m of core CAST-2. Palynostratigraphic data show a general homogeneity of the microbotanical record, apart from the lowermost sample (31.85 m), in which pollen and microcharcoal concentrations displayed the minimum values.

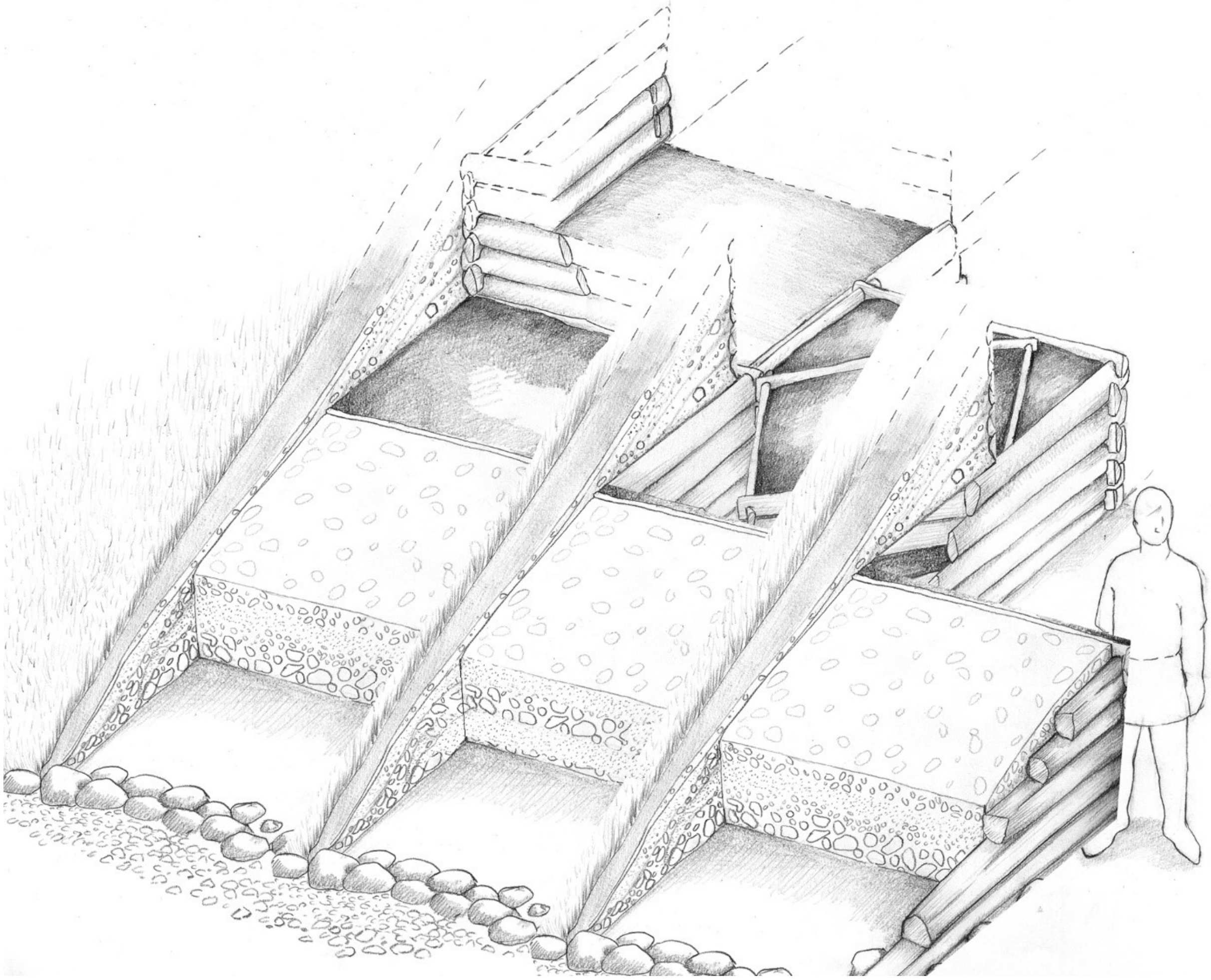

Figure S8. Reconstruction proposed by Vitri et al. (*46*) of the wooden “gabions” forming the structural framework of the rampart defending the castelliere of Udine in the later Bronze Age, as documented near Palazzo Mantica, at the southern toe of the mound (drawing by Federica Zendron). For location of the area see Figures 2, 4 and Supplementary Figure S2. The framework of vertical wooden buttress was filled by alternation of lenses of gravel and clays forming a structure of the earthen construction, but also allowing the movement of the rainy water.

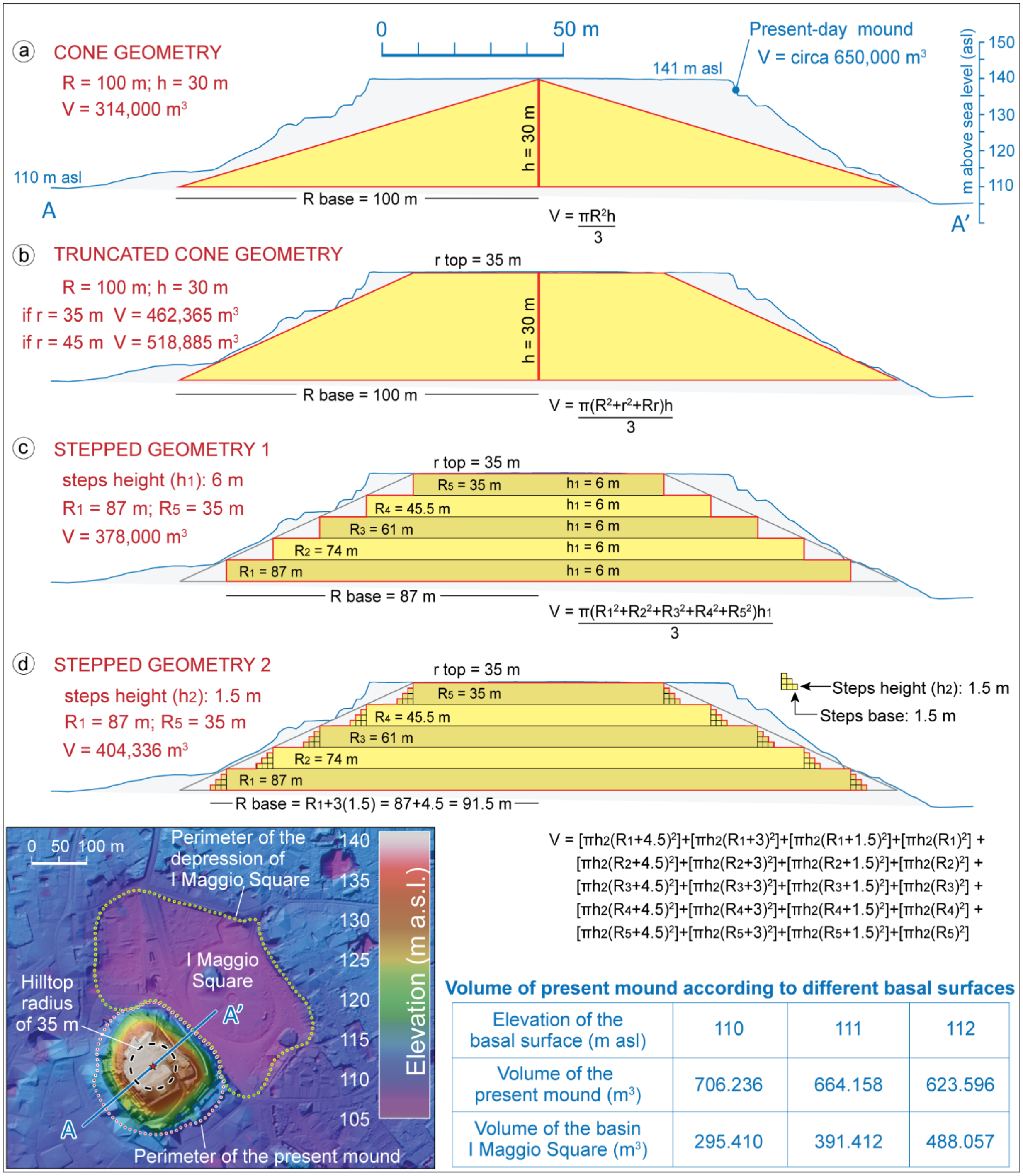

Figure S9. Estimation of the volume of the mound of Udine and comparison between the present and the prehistoric situation. Considering that the basal surface of the mound is at 110-111 m asl, currently the minimum volume of the anthropogenic hill is over 650,000 m^3. From the topographic depression corresponding to I Maggio Square, the estimated volume of quarried sediment ranges between 300,000 and 450,000 m^3. According to different simulations, the prehistoric mound had a minimum volume exceeding 350,000 m^3, but the most probable estimation is between 400,000 and 500,000 m^3. The map is a DEM produced with software QGIS (https://www.qgis.org).

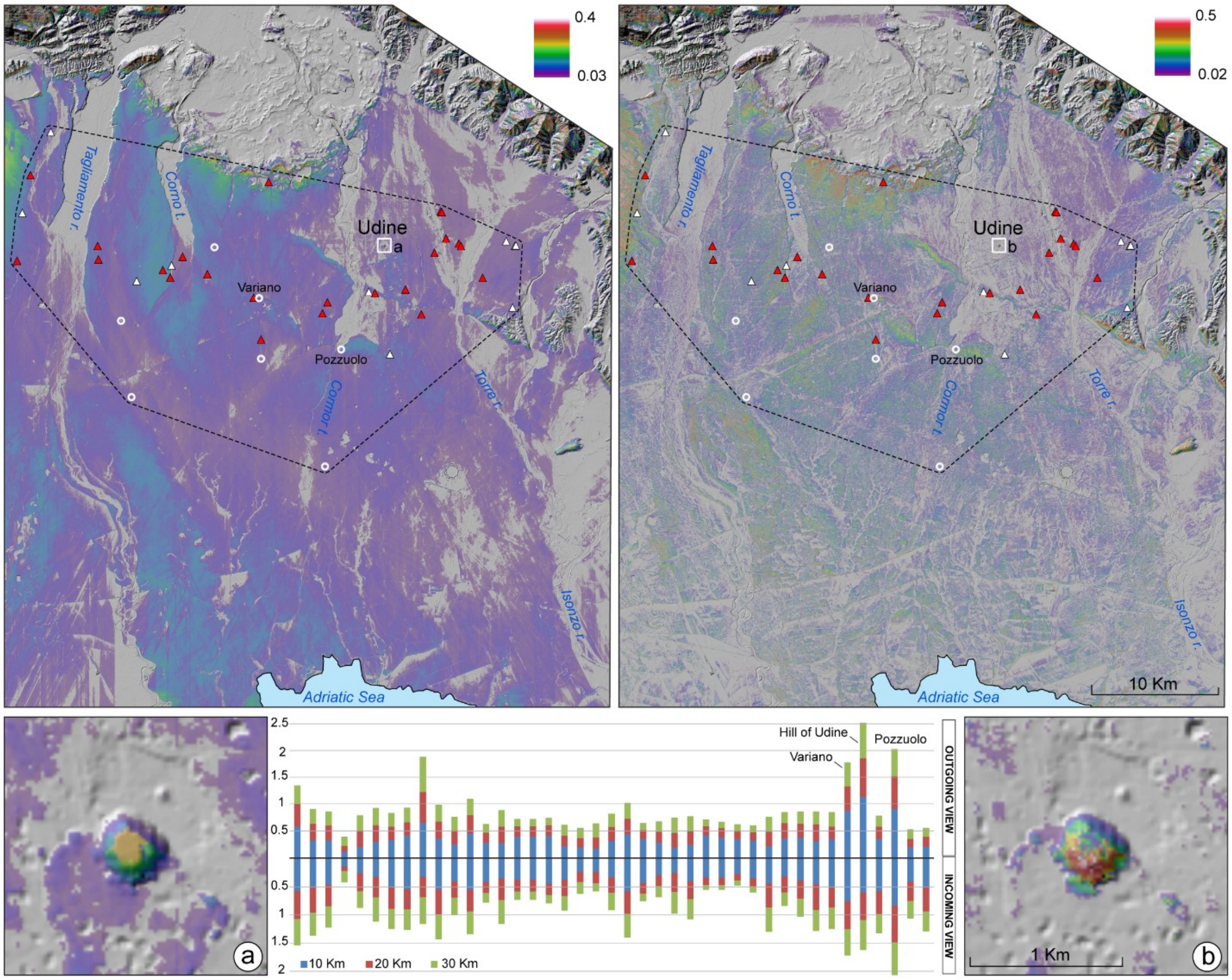

Figure S10. Total viewshed at a 30 km radius calculated for the central Friuli Plain between the Tagliamento and Isonzo rivers. Outgoing view (left) and incoming view (right) with details of the area of Udine Castle Hill for both views (a, b). The castellieri are indicated by white circles, definite tumuli by red triangles, alleged tumuli by white triangles, and the convex hull by the dotted line. The bar chart describes the visibility index for each of the 41 considered sites (castellieri and tumuli) at 10, 20 and 30 km radius. The maps were generated elaborating the DEMs with software QGIS (https://www.qgis.org) and Adobe Illustrator (www.adobe.com).

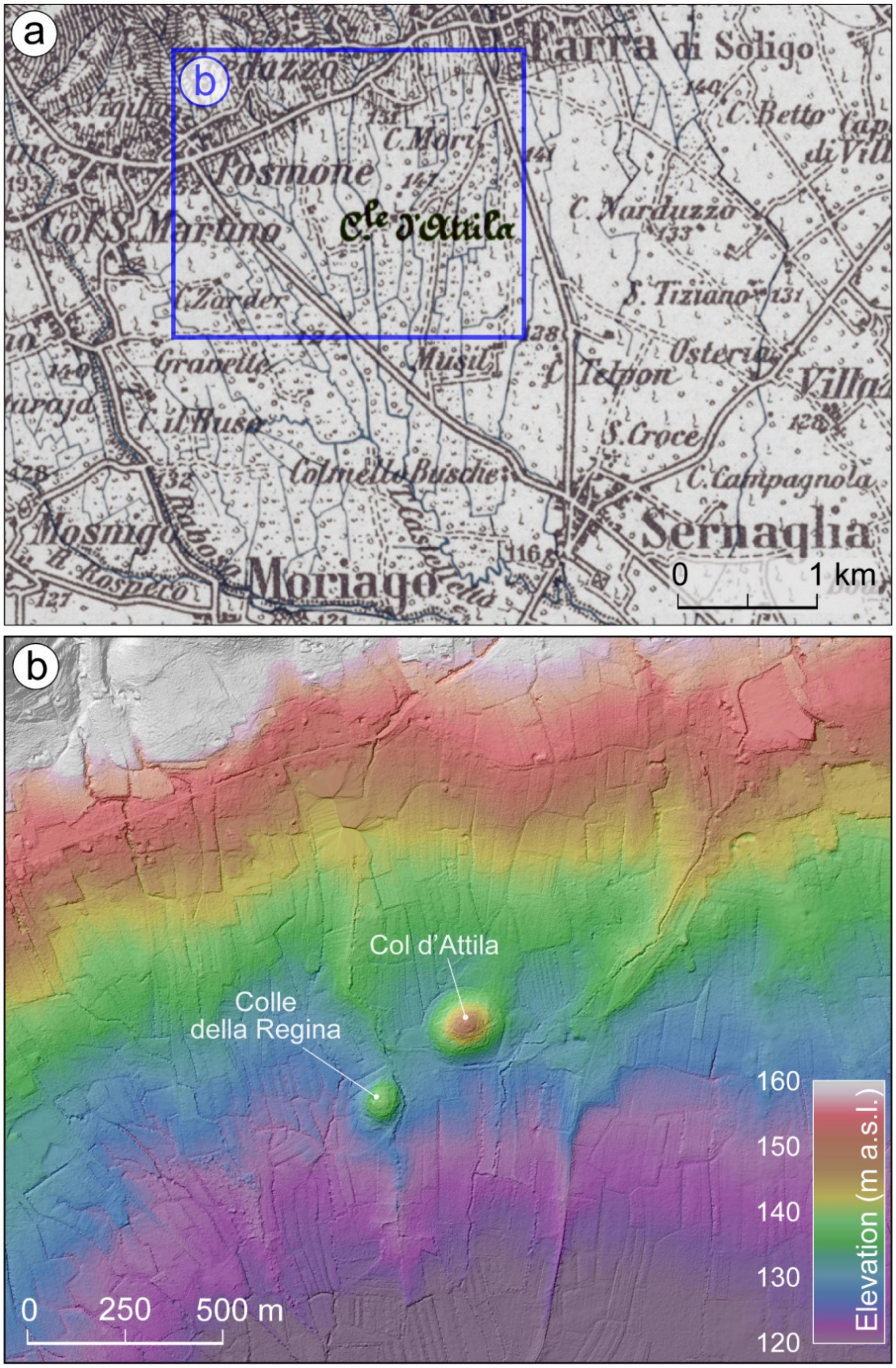

Figure S11. The isolated hill near Farra di Soligo named “Col d'Attila” (Hill of Attila). For location see Figure 1d. (a) Topographic map of the Austro-Hungarian Kingdom of 1908 at scale 1:75,000, with the name Colle d'Attila highlighted. (b) Together with the other relief of Colle della Regina (Hill of the Queen), these small and isolated hills are not sounding with the geological setting of the area and their origin is still debated. DTM elaborated from LiDAR data of the Province of Treviso with software QGIS (https://www.qgis.org) and Adobe Illustrator (www.adobe.com).

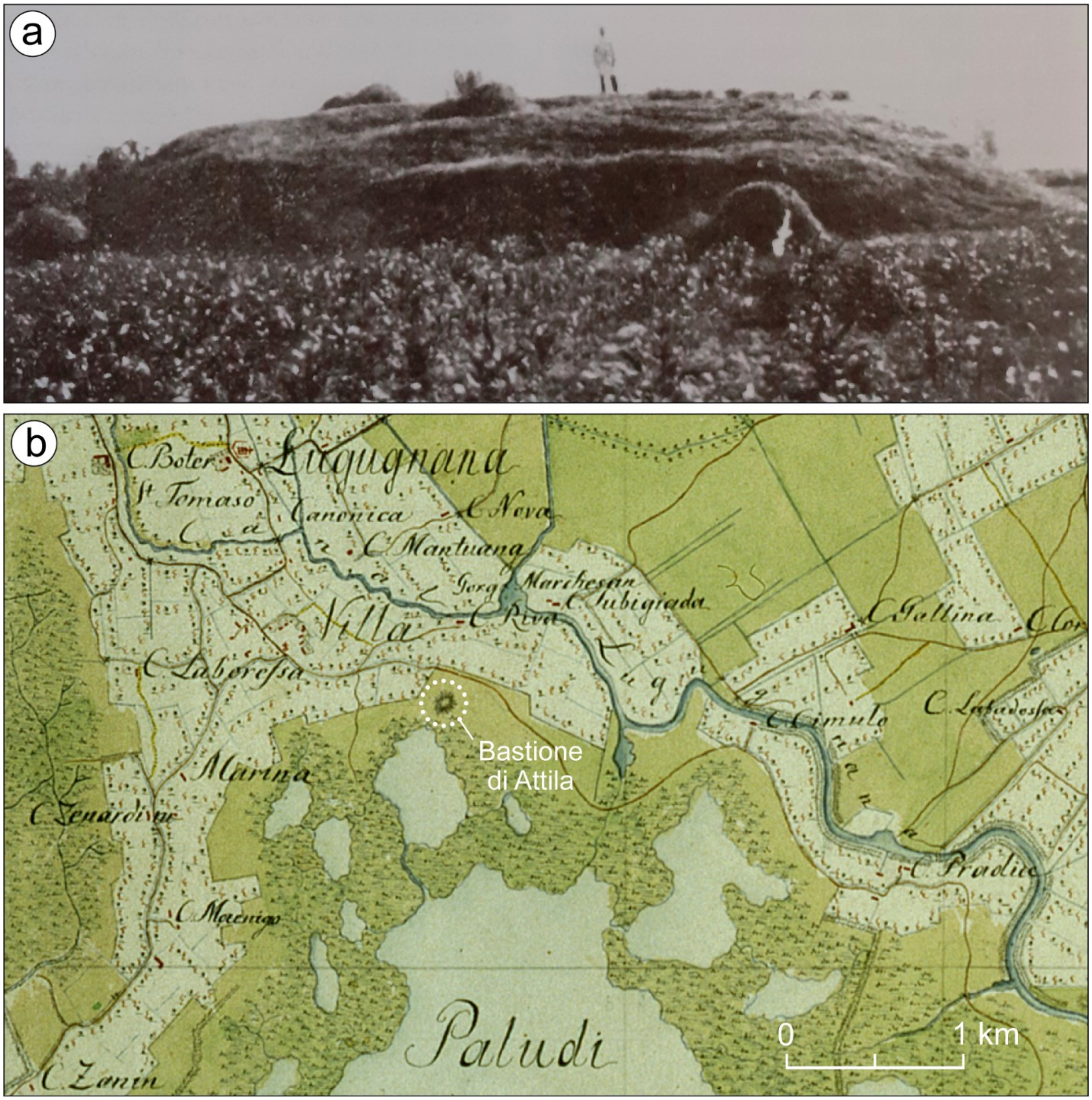

Figure S12. The mound of Lugugnana, locally called as the Bastione of Attila. The area is also named also "Mottaron" (the large mound in Italian). For the location see Figure 1d. a) Picture of the beginning of 20th century, just before the area was levelled; b) the topographic map surveyed by the Austro-Hungarian army between 1798 and 1805 [Kriegskarte of Anton von Zach; the characteristics of the map are described in (*59*)], with the indication of the mound. The description of the Bastione of Attila is also reported in the famous book "Le confessioni di un italiano" by Ippolito Nievo (*61*).

Phase	Sub-phase	Before Common Era (BCE)
Early Bronze Age (EBA)		ca. 2200–1650 BCE
Middle Bronze Age (MBA)		ca. 1650–1350/30 BCE
Recent Bronze Age (RBA)		ca. 1350/30–1150 BCE
	Recent Bronze Age 1 (RBA1)	ca. 1350/30–1200 BCE
	Recent Bronze Age 2 (RBA2)	ca. 1200–1150 BCE
Final Bronze Age (FBA)		ca. 1175/50–950 BCE
	Final Bronze Age 1(FBA1)	ca. 1175/50–1100
	Final Bronze Age 2 (FBA2)	ca. 1100–1000
	Final Bronze Age 3 (FBA3)	ca. 1000–950/925
Early Iron Age	Early Iron Age	ca. 950-550 BCE
Early Iron Age	Early Iron Age 1	ca. 950/925-800 BCE
	Early Iron Age 2	ca. 800-600 BCE

Table S1. Chronological framework of the reference Bronze Age in Northern Italy used in this research. The table is based on Pacciarelli (*62*), Cardarelli (*10*) and Borgna et al. (*11*).

Core Name	Coordinate (WGS84)	Elevation of the top (m asl)	Reached depth (m)	Depth of the base of historic deposits (m)	Depth of the base of Prehistoric mound (m)	Depth of top of natural deposits (m)
CAST-1	46° 3'53.69"N 13°14'6.92"E	141.2	20	6	-	-
CAST-2	46° 3'54.39"N 13°14'10.41"E	140.7	40	6.5	30.8	31.2
CAST-3	46° 3'53.61"N 13°14'6.28"E	133.2	25	3	21.3	23.6
CAST-4	46° 3'54.43" 13°14'7.79"	141.4	40	5	31.1	31.8
CAST-5	46° 3'52.34"N 13°14'8.38"E	140.8	40	1.5	29.0	29.0

Table S2. Location and main characteristics of the stratigraphic cores carried out between 2020 and 2022 from the top of the hill of Udine. The stratigraphic logs of the cores are described in Figure S6 and Data S1.
